# Characterization of Early Peripheral Immune Responses in Patients with Sepsis and Septic Shock

**DOI:** 10.3390/biomedicines10030525

**Published:** 2022-02-23

**Authors:** Jesús Beltrán-García, Rebeca Osca-Verdegal, Beatriz Jávega, Guadalupe Herrera, José-Enrique O’Connor, Eva García-López, Germán Casabó-Vallés, María Rodriguez-Gimillo, José Ferreres, Nieves Carbonell, Federico V. Pallardó, José Luis García-Giménez

**Affiliations:** 1 Center for Biomedical Research Network on Rare Diseases (CIBERER), Carlos III Health Institute, 46010 Valencia, Spain; jesus.beltran@ext.uv.es (J.B.-G.); rebeca.osca@ext.uv.es (R.O.-V.); e.maria.garcia@uv.es (E.G.-L.); federico.v.pallardo@uv.es (F.V.P.); 2 INCLIVA Biomedical Research Institute, 46010 Valencia, Spain; mariarodriguezgimillo@gmail.com (M.R.-G.); ferreresj@gmail.com (J.F.); edurnecarbonell@yahoo.es (N.C.); 3 Department of Physiology, Faculty of Medicine and Dentistry, University of Valencia, 46010 Valencia, Spain; 4 Laboratory of Cytomics, Joint Research Unit CIPF-UVEG, University of Valencia, 46010 Valencia, Spain; beatriz.javega@uv.es (B.J.); jose.e.oconnor@uv.es (J.-E.O.); 5 Flow Cytometry Unit, IIS INCLIVA, Fundación Investigación Hospital Clínico Valencia, 46010 Valencia, Spain; gherreramartin@gmail.com; 6 EpiDisease S.L. (Spin-Off CIBER-ISCIII), Parc Científic de la Universitat de València, 46980 Paterna, Spain; german.casabo@epidisease.com; 7 Intensive Care Unit, Clinical University Hospital of Valencia (HCUV), 46010 Valencia, Spain

**Keywords:** sepsis, immune response, innate immune system, adaptive immune system, cytokines, immune mediators, immunity mediated by endothelium

## Abstract

(1) Background: Sepsis is a life-threatening condition caused by an abnormal host response to infection that produces altered physiological responses causing tissue damage and can result in organ dysfunction and, in some cases, death. Although sepsis is characterized by a malfunction of the immune system leading to an altered immune response and immunosuppression, the high complexity of the pathophysiology of sepsis requires further investigation to characterize the immune response in sepsis and septic shock. (2) Methods: This study analyzes the immune-related responses occurring during the early stages of sepsis by comparing the amounts of cytokines, immune modulators and other endothelial mediators of a control group and three types of severe patients: critically ill non-septic patients, septic and septic shock patients. (3) Results: We showed that in the early stages of sepsis the innate immune system attempts to counteract infection, probably via neutrophils. Conversely, the adaptive immune system is not yet fully activated, either in septic or in septic shock patients. In addition, immunosuppressive responses and pro-coagulation signals are active in patients with septic shock. (4) Conclusions: The highest levels of IL-6 and pyroptosis-related cytokines (IL-18 and IL-1α) were found in septic shock patients, which correlated with D-dimer. Moreover, endothelial function may be affected as shown by the overexpression of adhesion molecules such as s-ICAM1 and E-Selectin during septic shock.

## 1. Introduction

Sepsis is a life-threatening condition that occurs as a consequence of a dysregulated host response to infection, which can evolve to tissue damage and organ dysfunction and thereby increase the risk of death (in-hospital mortality above 10%) [1,2]. Septic shock (SS) is a subtype of sepsis in which circulatory, cellular and metabolic abnormalities are associated with a greater risk of mortality than in sepsis alone (up to 40%) [1,2].

Sepsis is one of the leading causes of death worldwide, producing more deaths annually than prostate and breast cancer and human immunodeficiency virus/acquired immunodeficiency syndrome combined, with the numbers of cases increasing yearly [3]. Current epidemiologic studies estimate almost 50 million cases of sepsis around the world and up to 11 million deaths every year [4].

The response of the immune system during sepsis is a complex dynamic and time-dependent process. Cells of the innate immune system release high levels of pro-inflammatory cytokines triggering a “cytokine storm” and sometimes induce apoptosis of immune cells during the first hours of sepsis, leading to early death in septic patients [5]. In fact, the main function of pro-inflammatory cytokines is to protect the host from invading pathogens during the initial stages of infection. However, a compensatory anti-inflammatory response (also known as CARS) can result from the hyperinflammatory process, which is characterized by increased anti-inflammatory and decreased pro-inflammatory cytokine production. Indeed, CARS can sometimes occur simultaneously to systemic inflammatory response syndrome (SIRS), which may contribute to an immunosuppressive state in septic patients, contributing to death in the short-term [6,7]. It is of note that the exacerbated hyperinflammatory response produced can also cause damage in uninfected tissues and lead to the dysfunction of different organs and systems inducing hypotension, vasculature damage and clotting events, which finally contribute to the worsening of patients and death [8,9,10].

Nonetheless, a simultaneous release of pro- and anti-inflammatory mediators which contribute to the deregulation of the patient’s immune system, and even produce immunosuppression, refs. [11,12,13] can take place. Exacerbation of any of these complex and heterogeneous responses can reduce the probability of survival of septic patients, compromise their long-term health status and increase their risk of death by serious morbidities (i.e., acute lung injury, cardiovascular problems or neurological deficits, among others). In fact, persistent activation of the innate immunity and dysregulation of adaptive immune responses results in chronic immunosuppression accompanied by chronic inflammation [14,15]. Moreover, immunosuppression increases susceptibility to secondary infections, which are associated with 13% of deaths related to sepsis [8,14,15]. It is noteworthy that a subset of patients with chronic critical illness (CCI) due to comorbidities and usually dependent on mechanical ventilation do not survive after months of unsuccessful treatment [16]. A new syndrome of multiorgan failure has been described among some patients discharged alive from intensive care units (ICU), mainly in patients with CCI. This syndrome has been coined “persistent inflammatory, immunosuppression, catabolic syndrome (PICS)” and is considered a new multiorgan dysfunction phenotype caused by sepsis [5,17]. Interestingly, although the mechanisms underlying PICS have not yet been completely clarified, the interaction between elevated C-reactive protein (CRP) and myeloid-derived suppressor cells, and increased levels of inflammatory cytokines (such as interleukin (IL)-6 and IL-8), is required for the development of PICS [15,18]. Moreover, the immunosuppressed state is also characterized by the production of inhibitory cytokines such as IL-10 and IL-4, which limit the intensity of immune cell response and negatively modulate hyper-inflammatory responses. In this regard, some authors have proposed the role of cytokines and other pro- and anti-inflammatory mediators as biomarkers during sepsis and septic shock. Several studies have focused on establishing the role of cytokines as biomarkers of disease progression including estimation of death risk. As result, some of these cytokines, such as IL-1, IL-6, IL-8 or MCP-1, among others, have been postulated as predictive values for sepsis severity and progression [19]. However, most of them, particularly when considered alone, showed insufficient specificity and sensitivity to predict the disease progression in larger clinical trials [19], even though the initial attempts on drug development mainly focused on blocking inflammation and have not proven any tangible outcome [20].

The aim of the present study was to simultaneously characterize a wide array of cytokines and mediators participating in the immune response during the first stages of sepsis, particularly in septic and septic shock patients. This approach may help us to identify different molecular mechanisms and provide a picture about how the immune system acts during the first stages of sepsis progression. We present the molecular events leading to the initial immune response and how it is regulated in septic and septic shock patients. Furthermore, monitoring changes in the clinical signatures of immune cells could represent a promising tool to detect individuals at risk of immunosuppression and suggest new therapeutic pathways for controlling the immune impairment present in septic patients.

## 2. Materials and Methods

### 2.1. Patient Selection

The Clinical University Hospital of Valencia (HCUV), Spain, has a sixteen-bed medical ICU where patients were enrolled. Thirty non-consecutive patients admitted at the ICU with the diagnosis of spontaneous intracranial hemorrhage who were established as non-septic ICU controls (*n* = 5), sepsis (*n* = 10) and septic shock (*n* = 15), were included in the study. All the septic patients met the Sepsis-3 Consensus definition criteria [1] and they also had a community-acquired origin. The collection of these non-consecutive patients was obtained throughout 2017 (January to December). Non-septic ICU controls were collected from February 2017 to July 2018. Septic patients presented an acute change in total SOFA score of ≥2 points consequent to the infection and septic shock, and they meet the criteria of patients requiring vasopressors to maintain MAP > 65 and having serum lactate > 2 mmol/L despite adequate volume resuscitation. We decided to include another homogeneous medical non-septic pathology as a possible control group. However, sepsis from a nosocomial origin was excluded in order to avoid immunity modulatory factors linked to prolonged hospital admission. Patients participating in this study were enrolled during the first 6 h after admission in the ICU when a suspicion of sepsis and septic shock existed. Blood samples were obtained by artherial catheter after ICU admittance in K_2_EDTA (BD Vacutainer^®^, (Franklin Lakes, NY, USA), ref 367861) and citrate tubes (BD Vacutainer^®^ Citrate, Ref 363080) from all patients. Immediately, blood samples were sent to INCLIVA’s Biobank to be processed and separate plasma from blood cell fractions. After processing, aliquots of plasma samples and blood cell fractions were immediately frozen to −80 °C, registered and stored at INCLIVA’s biobank (Biomedical Research Institute INCLIVA, Valencia, Spain). Retrospectively, cases who met SEPSIS-3 criteria for sepsis and septic shock definitions were selected for the analysis and plasma samples obtained from the INCLIVA’s biobank to perform the experiments.

The amounts of cytokines, immune modulators and other endothelial mediators were analyzed in these samples and compared among the three types of critically ill patients. Plasma samples from 10 healthy (male–female ratio 5:5) subjects were analyzed as well (Table 1).

Informed consent was obtained from each participant. All the experimental protocols and methods were performed after obtaining approval from the HCUV’s Biomedical Research Ethics Committee. All the procedures were performed according to relevant international guidelines and regulations.

### 2.2. Estimation of Immune Cell Composition

Retrospectively, the total DNA of 16 patients (critically ill non-septic patients (*n* = 4), septic patients (*n* = 7) and SS patients (*n* = 5) was isolated from the total blood cell pellet stored at the INCLIVA biobank with the All-In-One DNA/RNA Miniprep Kit (BS88203, Bio Basic Canada Inc (Markham, ON, Canada)) following the manufacturer’s instructions. Purified DNA was quantified with the fluorometric method (Quant-iT PicoGreen dsDNA Assay, Life Technologies, Carlsbad, CA, USA). Genome-wide methylation was assessed as previously described by Pozo-Lorente et al. [21] using Infinium Human DNA Methylation EPIC 850 K arrays (Illumina Inc., San Diego, CA, USA) scanned on an Illumina HiScan SQ scanner (Illumina Inc., San Diego, CA, USA). The minfi R-package [22] was used to process and normalize the arrays [22,23].

The proportions of the different immune cells were obtained using the estimate Cell Counts 2() function of the FlowSorted.Blood.Epic package [24] using a process of deconvolution (Identifying Optimal Libraries—IDOL) based on discriminating differentially methylated regions (DMRs) specific to each cell type [25]. The neutrophil-to-lymphocyte ratio (NLR) was calculated taking the ratio between the estimated cell proportion of neutrophils and the estimated cell proportions of CD4T, CD8T, NK and B cells.

### 2.3. Measurement of Cytokines in Plasma from Patients in Intensive Care Units

Plasma levels of IL-1α, IL-1β, IL-12p70, interferon (IFN)- α, IFNγ-α, IL-6, IL-8, IL-10, tumor necrosis factor (TNF-α, granulocyte-macrophage colony-stimulating factor (GM-CSF), macrophage inflammatory protein 1α (MIP-1α, MIP-1β, monocyte chemoattractant protein-1 (MCP-1), E-selectin, P-selectin, intercellular adhesion molecule 1 (si-ICAM1) (IL-4, IL-13, IL-17 and interferon-γ-inducible protein 10 (IP-10) in samples from healthy individuals (*n* = 10), critically ill non-septic patients (*n* = 5) and patients with sepsis (*n* = 10) and SS (*n* = 15) were measured with the Human Inflammation 20-plex ProcartaPlex Panel (ThermoFisher, Waltham, MA, USA), according to the manufacturer’s instructions. Briefly, 25 μL of plasma were incubated with 50 μL of beads overnight. Afterwards beads were washed and incubated with antibodies for 30 min and then with streptavidin-PE for additional 30 min. When finalized beads were incubated with 120 μL of reading buffer and data were acquired on Luminex Magpix Instrument (Luminex Corporation, Austin, TX, USA). The detection limit was indicated in the manufacturer’s manual.

Human IL-18 levels were measured in triplicate in 50 μL of plasma using the Human IL-18 ELISA (Thermo Fisher, Waltham, MA, USA). IL-27 in 50 μL of plasma from sepsis and septic shock patients was measured using the Human IL-27 ELISA kit (Thermo Fisher, Waltham, MA, USA), according to the manufacturer’s instructions. S100A8 and S100A9 were measured in 50 μL of plasma with the Human S100A8 ELISA (Thermo Fisher, Waltham, MA, USA) and Human S100A9 ELISA kits (MyBioSource, San Diego, CA, USA), according to the manufacturer’s instructions. The detection limit was indicated in the manufacturer’s manual.

### 2.4. Statistical Analysis

The statistical analyses were performed with SPPS v23, and Prism software (Software Inc., San Diego, CA, USA) was used for graphics. Values are expressed as the median with interquartile ranges.

The normality of samples was determined with the Kolmogorov–Smirnov normality test, and samples did not follow a Gaussian distribution. The non-parametric Mann–Whitney test was used to analyze differences between two non-paired groups with a significance level of 0.05. The Kruskal–Wallis test was used to analyze differences among ICU controls, sepsis and SS patients, followed by a post hoc test using Bonferroni correction for α (0.05/3) when we compared clinical data. For comparisons in cytokine levels in different groups we performed a Kruskal-Wallis test followed by Dunns’ post hoc test. Spearman’s analysis was used for the correlation analysis among variables and the *p*-values were adjusted for multiple comparisons by the Benjamini–Hochberg method with the function p.adjust() from the stats package. An FDR < 0.1 was considered significant.

The R version 4.0.0 was used for clustering analysis and preparation of the heatmap. Hierarchical clustering was performed with the function hclust() from the stats package, and the heatmap was generated using the function heatmap.2() from the gplots package.

## 3. Results

### 3.1. Immune Cell Proportions Are Different among Critically Ill Non-Septic Patients, Sepsis and Septic Shock Patients

**Figure 1:**
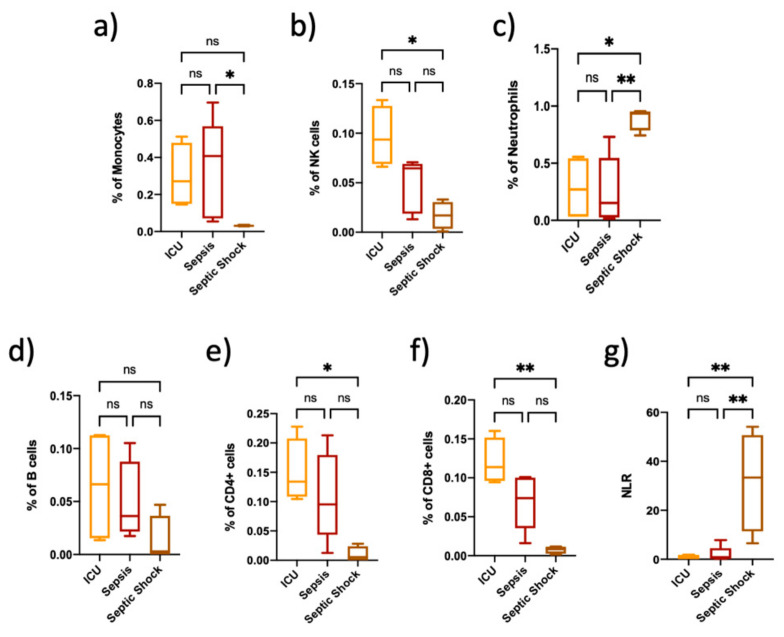
Estimation of the immune sub-population in critically ill, septic and septic shock patients; (**a**–**f**) Immune cell subpopulations estimated using a process of deconvolution based on discriminating differentially methylated regions (DMRs) specific to each cell type; (**g**) Neutrophil to lymphocyte count ratio (NLR) in ICU, sepsis and septic shock patients. Estimates were obtained from DNA methylation data from critically ill non-septic patients (*n* = 4), septic (*n* = 7) and septic shock (*n* = 4) patients. Data are expressed as mean ± SEM). ns = non-significant. *p* value; * *p* < 0.05; ** *p* < 0.01. The lines at the top of the box plots indicate differences between compared conditions.

Immune cell proportions estimated from DNA methylation data showed differences among groups. Septic shock patients showed increased levels of neutrophils and low lymphocyte populations compared to septic and critically ill patients (Figure 1). We also performed an analysis of the NLR because it has shown to be useful as a diagnostic and prognostic marker in patients with sepsis [26]. Our results showed a high NLR ratio in SS patients compared to the other groups of patients (Figure 1). Moreover, we looked for the white blood cell and platelet counts in the clinical records and observed that polymorphonuclear cells were increased in sepsis and septic shock patients when compared to critically ill non-septic patients. Conversely, platelets were decreased in sepsis and SS when compared to other patients (Figure S1).

### 3.2. Enhancement of Pyroptosis-Related Interleukins in Sepsis and Septic Shock Patients

**Figure 2:**
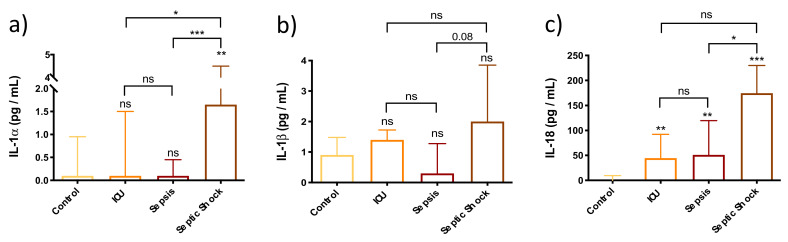
Bar graph (Median ± IQR) of circulating levels of pyroptosis-related cytokines in control group, ICU, sepsis and septic shock patients. (**a**) Circulating levels of IL-1α; (**b**) Circulating levels of IL-1β; (**c**) Circulating levels of IL-18. Each sample was measured in duplicate. Groups were compared by the Kruskal–Wallis test with post hoc Dunn’s multiple comparison test. ns = non-significant. *p* value; * *p* < 0.05; ** *p* < 0.01; *** *p* < 0.001. The lines at the top of the box plots indicate differences between compared conditions. The number of subjects analyzed were as follows: control (*n* = 10); intensive care unit (ICU) (*n* = 5); sepsis (*n*= 10); septic shock (SS) (*n* = 15).

The results obtained in Figure 2 demonstrate that SS patients have significantly elevated circulating levels of both IL-1α and IL-18 compared to septic patients and the control group. Likewise, SS patients showed higher IL-1α levels than the ICU group. However, there were no statistically significant differences in circulating IL-1β levels among the different groups.

### 3.3. Innate Immunity Is Overactivated in Septic Shock Patients at Early Stages of the Septic Episode

**Figure 3:**
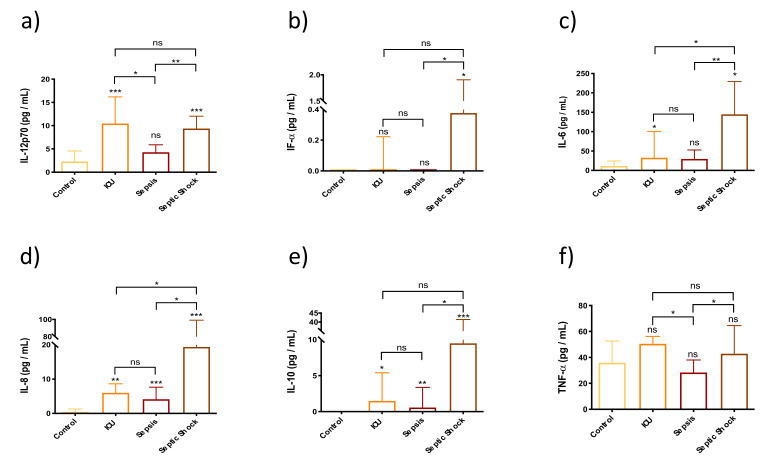
Bar graph (Median ± IQR) of circulating levels of cytokines activating macrophages and NK cells, in control group, ICU, sepsis and septic shock patients. (**a**) Circulating levels of IL-12p70; (**b**) Circulating levels of IFN-α; (**c**) Circulating levels of IL-6; (**d**) Circulating levels of IL-8; (**e**) Circulating levels of IL-10; (**f**) Circulating levels of TNFα. Each sample was measured in duplicate. ns = non-significant *p* value; * *p* < 0.05; ** *p* < 0.01; *** *p* < 0.001. The lines at the top of the box plots indicate differences between compared conditions. The number of subjects analyzed were as follows: control (*n* = 10); intensive care unit (ICU) (*n* = 5); sepsis (*n* = 10); septic shock (SS) (*n* = 15).

Figure 3 shows that SS patients had elevated circulating levels of pro-inflammatory cytokines IL-12p70, IFN-α, IL-6 and IL-8 compared to both the control group and the group of septic patients. However, the patients belonging to the non-septic ICU group did not show differences in the levels of these cytokines when compared to the SS group.

These results indicate that SS patients had higher levels of pro-inflammatory IL-6, IL-8 and anti-inflammatory IL-10 than the control group, the non-septic ICU patients and the septic patients. However, SS patients did not overexpress TNF-α compared to both control groups, although the levels were higher in SS compared to sepsis. Furthermore, septic patients also showed elevated levels of IL-8 and IL-10 compared to the control group, and lower levels of TNF-α compared to the non-septic ICU group. Although not shown in this figure, we also measured the levels of IFN-γ and found that SS patients’ group was the only group in which we were able to detect this cytokine (2.073 ± 3.20 pg/mL).

### 3.4. Chemokines Are Overexpressed in Plasma from Septic Shock Patients, Thereby Altering Endothelial-Mediated Immune Response

**Figure 4:**
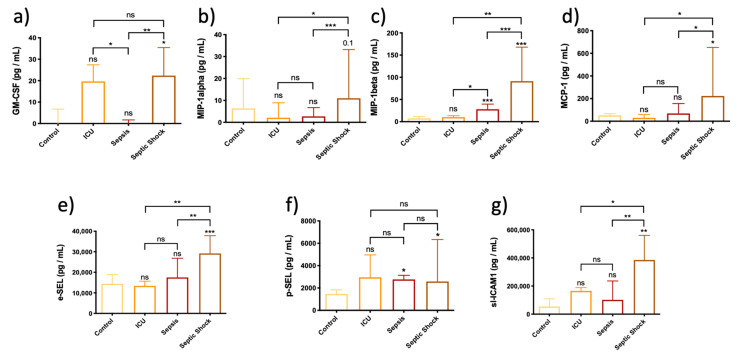
Bar graph (Median ± IQR) of circulating levels of monocyte and neutrophil mediators in control, ICU, sepsis and septic shock patients. (**a**) Circulating levels of GM-CSF; (**b**) Circulating levels of MIP-1α; (**c**) Circulating levels of MIP-1β; (**d**) Circulating levels of MCP1; (**e**) Circulating levels of e-SEL; (**f**) Circulating levels of p-SEL; (**g**) Circulating levels of s-ICAM1. Each sample was measured in duplicate. ns = non-significant *p* value; * *p* < 0.05; ** *p* < 0.01; *** *p* < 0.001. The lines at the top of the box plots indicate differences between compared conditions. The number of subjects analyzed were as follows: control (*n* = 10); intensive care unit (ICU) (*n* = 5); sepsis (*n* = 10); septic shock (SS) (*n* = 15).

The results in Figure 4 show that SS patients had the highest levels of chemokines GM-CSF, MIP-1α and MIP-1β and MCP-1 compared to the control, ICU and sepsis groups.

The results presented in Figure 4 indicate that the SS group showed the highest plasma levels of E-selectin and ICAM1 compared to the control, ICU and sepsis groups. In combination with the ratios of immune cell subsets, these results suggest that the most stimulated response in SS patients is mediated by neutrophils, thereby promoting neutrophilic inflammation in the endothelium, since both chemokines and endothelial adhesion molecules were increased in these patients.

### 3.5. Adaptive Immune Response Is Not Fully Functional in the First Stages of Septic Shock Development

**Figure 5:**
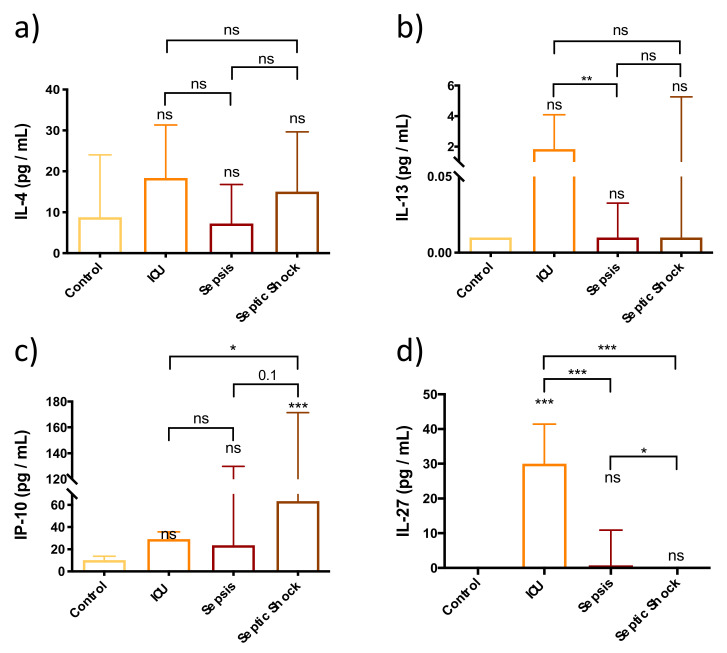
Bar graph (Median ± IQR) of circulating levels of adaptive immune mediators in control, ICU, sepsis and septic shock patients. (**a**) Circulating levels of IL-4; (**b**) Circulating levels of IL-13; (**c**) Circulating levels of IP-10; (**d**) Circulating levels of IL-27. Each sample was measured in duplicate. ns = non-significant *p* value; * *p* < 0.05; ** *p* < 0.01; *** *p* < 0.001. The lines at the top of the box plots indicate differences between compared conditions. The number of subjects analyzed were as follows: control (*n* = 10); intensive care unit (ICU) (*n* = 5); sepsis (*n* = 10); septic shock (SS) (*n* = 15).

The results presented in Figure 5 show no differences in IL-4 and IL-13 levels in patients with SS compared to the control, ICU and sepsis groups. Regarding IL-27, the SS group showed lower levels of circulating IL-27 than the ICU and sepsis groups. The SS group had the highest plasma levels of IP-10 among all the groups studied. Interestingly, non-septic ICU patients showed higher plasma levels of IL-13 and IL-27 than the sepsis and the SS groups.

### 3.6. Immunomodulators Are Strongly Overexpressed in Septic Shock Patients

**Figure 6:**
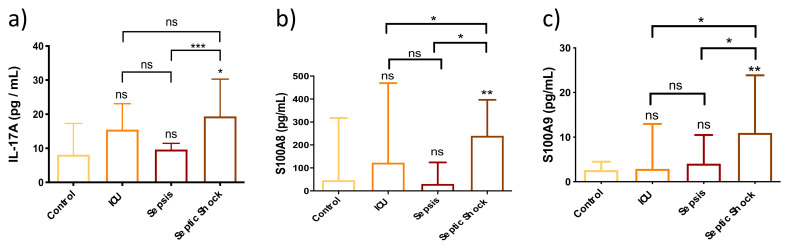
Bar graph (Median ± IQR) of circulating levels of different immunomodulators in control, ICU, sepsis and septic shock patients. (**a**) Circulating levels of IL-17A; (**b**) Circulating levels of S100A8; (**c**) Circulating levels of S100A9. Each sample was measured in duplicate. ns = non-significant *p* value; * *p* < 0.05; ** *p* < 0.01; *** *p* < 0.001. The lines at the top of the box plots indicate differences between compared conditions. The number of subjects analyzed were as follows: control (*n* = 10); intensive care unit (ICU) (*n* = 5); sepsis (*n* = 10); septic shock (SS) (*n* = 15).

Our results showed the highest levels of IL-17A, S100A8 and S100A9 in the SS patient group compared to healthy control subjects, and non-septic ICU and septic patients (Figure 6). The overexpression of these factors linked to the increase of this cellular subset may contribute to neutrophilic inflammation in patients with SS.

### 3.7. Correlations of Different Cytokines and Immune Mediators

**Figure 7:**
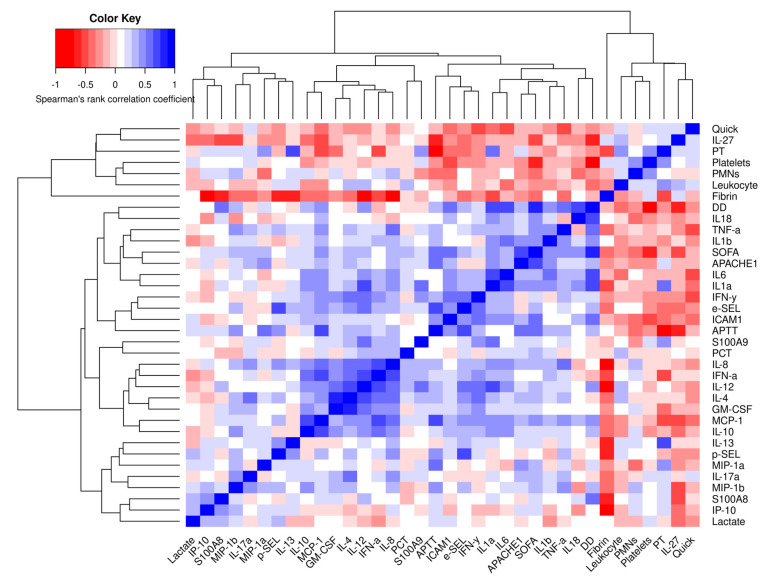
Hierarchical cluster representing the Spearman’s rank correlation coefficients (−1 to +1) among the clinical and analytical variables measured. Red color indicates a negative correlation and blue color indicates a positive correlation between compared parameters. The dendrograms were obtained by hierarchical clustering using the complete linkage method. Clusters of the dendrograms include cytokines, reactants, endothelial mediators and clinical features with a strong positive correlation. APACHE II: Acute Physiology and Chronic Health disease Classification System II; SOFA: Sequential [Sepsis-related] Organ Failure Assessment; APTT: activated partial thromboplastin time; DD: D-Dimer; PT: prothrombin time; PMNS: polymorphonuclear cells. The number of subjects analyzed were as follows: control (*n* = 10); intensive care unit (ICU) (*n* = 5); sepsis (*n* = 10); septic shock (SS) (*n* = 15).

We provide a series of correlation analyses among clinical parameters and the aforementioned cytokines and immune mediators relevant to sepsis and SS immune response. The Spearman’s rank correlation coefficients were calculated for cytokines, immune mediators and clinical parameters in cases (including sepsis and SS patients) and controls (including healthy subjects and ICU patients). The statistical values (correlation coefficient, *p*-value and adj.*p*-value) are shown in Supplementary Tables S1–S3, respectively. A hierarchical cluster using the complete linkage method was performed to group the variables analyzed and identify clusters of cytokines, endothelial mediators and clinical features with a positive correlation (Figure 7).

Several positive correlations were found between the severity clinical scores and markers with the different cytokines and immune mediators. In this regard, the APACHE and SOFA scores showed a positive correlation with the inflammatory cytokine IL-1α (Spearman r 0.412 and 0.471, respectively) and IL-1 β (Spearman r 0.438 and 0.429, respectively). Moreover, cytokine IL-1α appeared in the same cluster as pro-inflammatory cytokine IL-6 and both showed a significant strong correlation (Spearman r 0.745). Importantly, the relationship among these variables was clearly identified in a hierarchical cluster in Figure 7. In this cluster, pyroptosis-related cytokines IL-1α, IL-1β and IL-18 were found together D-dimer (DD). In this regard, cytokines IL-1α, IL-1β and IL-18 were positively correlated with DD (Spearman r 0.647, 0.509 and 0.727, respectively), thus showing a relationship between pyroptosis and a relevant marker of the common pathway of the coagulation cascade. Moreover, our results also showed how these cytokines released after activation of the inflammasome and initiation of pyroptosis showed a moderate correlation with endothelial mediators s-ICAM1 and E-selectin, indicating the implication of pyroptosis with the alteration of the endothelial function. In addition, s-ICAM1 positively correlated with E-selectin (Spearman r 0.512) which are both related with the adhesion of the leukocytes to the endothelium at the site of infection.

We also found a moderate positive correlation between anti-inflammatory cytokine IL-10 and MCP-1 (Spearman r 0.674). MCP-1 plays a role in reducing inflammation via the production of anti-inflammatory cytokines, such as IL-10. Importantly, MCP-1 also showed a moderate correlation with DD (Spearman r 0.501). This cluster composed by pyroptosis-related cytokines (IL-1α, IL-1 β and IL-18, anti-inflammatory mediators (IL-10 and MCP-1) and DD suggests that both, the inflammation and the release of anti-inflammatory cytokines, may occur simultaneously and be related to a pro-coagulation phenotype, which in turn seriously contributes to increase SOFA punctuation. In fact, DD showed a strong positive correlation with SOFA (Spearman r 0.819). Interestingly, we also found that IL-1α showed a positive correlation with mortality in septic shock patients (Spearman r 0.559).

Other cluster was found to be formed by IL-8, IFN-α, IL-12, IL-4, GM-CSF, and the anti-inflammatory mediators MCP-1 and IL-10. Among them, a strong positive correlation was found between IL-8 and IFN-α (Spearman r 0.743), IL-4 with GM-CSF (Spearman r 0.786) and IL-12 (Spearman r 0.685). IL-12 also was correlated with the pyroptsis-related cytokine IL-1α (Spearman r 0.647), although both cytokines were not in the same cluster. Moreover, MCP-1 showed a positive correlation with IFN-α (Spearman r 0.645). In addition, IL-12 showed a positive correlation with both IFN-α (Spearman r 0.678).

Interestingly, IL-4 showed a moderate correlation with IL-17A (Spearman r 0.532). In this regard, Th2-derived IL-4 and Th17-derived IL-17A may generate a chronic inflammatory milieu in critically ill patients. As shown previously, GM-CSF was included in the same cluster as IL-4 since both T-helper cells, Th2 and Th17, can release this factor which is responsible for stimulating stem cells to produce granulocytes such as neutrophils and monocytes (Figure S2).

## 4. Discussion

Sepsis is a very worrisome syndrome characterized by a dysregulated host immune response to infection, mediated by an initial hyperinflammatory phase followed by anti-inflammatory response, which can sometimes coexist and induce, in many cases, permanent immunosuppression. In particular, SS is the worst clinical phenotype and is characterized by organ dysfunction and strong immunosuppression, increasing the risk of death. Characterization of the innate and adaptive immune responses mediated in septic and SS patients may unveil the complex events that occur in sepsis and will help to develop new therapies to improve the diagnosis, prognosis and treatment of sepsis [5].

Among the inflammatory-related processes, pyroptosis is a form of programmed cell death associated with pro-inflammatory phenotypes occurring at early stages of infection [27]. In general, pyroptosis protects host organisms against pathogens, such as bacteria, viruses, protozoa or fungi, by producing interleukins IL-1α, IL-1β and IL-18, but pyroptosis in immune cells and endothelial cells can also induce cellular toxicity and tissue damage in the host [28]. IL-1α and IL-1β play an important role in inflammation and vasodilation, but IL-18 is also involved in immune regulation by promoting Th1 cell activation and enhancing the cytotoxic activity of CD8^+^ T cells [29] and NK regulation. Importantly, NK are able of recognizing infected and stressed cells and respond by killing them and secreting IFN-γ, a macrophage-activating cytokine. Likewise, macrophages ingest microbes and produce IL-12, which activates NK lymphocytes to secrete more IFN-γ, thus increasing their activity to each other. In addition, other molecules such as type-1 interferons (e.g., IFN-α) enhance the activity and efficacy of NK cells by increasing their microbicidal capacity [30]. In addition, NK cells are one of the first targets of IL-18, which cooperates with signal transductors and cytokine activators, such as IL-12, to activate NK effector functions, in terms of IFN-γ production, cytotoxicity and expression of the Fas ligand [31,32]. Interestingly, in this work we found a correlation between IL-1α and IL-12 (Spearman r 0.647), and IL-12 and IFN-γ (Spearman r 0.576).

Importantly, IL-6 is secreted by macrophages in response to specific microbial molecules. This interleukin is responsible for stimulating acute phase protein synthesis and the production of neutrophils in the bone marrow. Moreover, IL-6 promotes the proliferation of B cells and increases the responses mediated by the pro-inflammatory T-helper 17 cells (Th17) and suppresses the function of anti-inflammatory regulatory T cells (Tregs) [33]. Interestingly, the highest levels of IL-6 were found in SS patients, just in those patients in which neutrophiles were elevated when compared to other cell types. In addition, one of the sources of pyroptosis-related cytokines (IL-18 and IL-1α) in SS patients can also be neutrophils, see Figure 1. These findings may reinforce the idea that neutrophils may act as a double-edged sword in severe sepsis [34], particularly in SS (severe phenotypes). Moreover, we cannot rule out the participation of other sources of IL-1α and IL18 since pyroptosis can also occur in endothelial cells which can be injured during septic shock [35,36].

Noteworthy, cytokines IL-1α, IL-1β and IL18 were found in a cluster which also included the APACHE and SOFA scores, showing a correlation among them. Importantly, IL-1α showed a strong correlation with cytokine IL-6, which indicates that both mediators may generate a pro-inflammatory state during the first hours of the septic episode. Additionally, pyroptosis-related cytokines positively correlated with s-ICAM1, and it was found in the same cluster as pyroptosis-related cytokines and endothelial mediators, indicating that IL-1α, IL-1β and IL18 contribute to a systemic hyperinflammatory state and the activation of the endothelium (Figure 7 and Tables S1–S3). Additionally, cytokines released after inflammasome activation and pyroptosis activation were also included in the same cluster and showed a moderate positive correlation with SOFA and strong correlation with DD, confirming that pyroptosis mediates the worsening of septic patients by contributing to coagulation and thrombus formation, as described previously [37,38].

**Figure 8:**
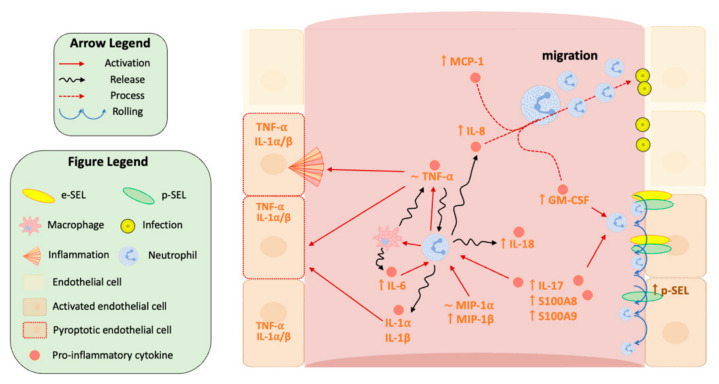
Graphical scheme of neutrophil immune response in septic shock patients. The upper arrow indicates overexpression; the lower arrow indicates down-regulation, ∼ indicates no variation in comparison to control samples. Orange names are pro-inflammatory cytokines, green names are anti-inflammatory cytokines, blue names are cells. A complete scheme is presented in Supplementary Materials (Figure S2).

Our results indicate that pyroptosis is an important player in the orchestration of innate immune response at least in the first stages of infection, contributing to adverse phenotypes that occur during the pathophysiology of sepsis (Figure 8). IL-17A is a pro-inflammatory cytokine produced by Th17 cells which induces the release of many cytokines (such as IL-6, G-CSF, GM-CSF, IL-1β, TGF-β, TNF-α) and chemokines (including IL-8, GRO-α and MCP-1) [39,40], with the final goal of increasing inflammation, the generation of granulocytes by bone marrow and attracting leukocytes to the site of infection [39,41,42]. Furthermore, IL-17A also induces TNF-α expression in macrophages [43], which is a pro-inflammatory cytokine involved in the recruitment and stimulation of neutrophils and monocytes. When macrophages detect pathogens, they release TNF-α in order to alert other cells of the immune system and other tissues, leading to inflammation. Thus, TNF-α is involved in systemic inflammation and is one of the cytokines that initiate the acute phase reaction.

Our results showed that IL-17A is highly expressed in the first stages of sepsis, specifically in SS patients. Furthermore, IL-17A showed a positive correlation with IL-4, and IL-4 was included in the same cluster as GM-CSF, as well as with MIP-1B (Tables S1–S3). These results indicate that IL-17A plays a prominent role in the activation and function of macrophages and neutrophil-mediated immunity by stimulating neutrophil production of pro-inflammatory molecules, including TNF-α, IL-1α and IL-4 [44]. TNF-α and IL-1α showed a moderate positive correlation and were highly expressed in septic shock patients, which may contribute to the activation of the endothelium by IL-1 and TNF-α, producing, among others, the expression of strong adhesion molecules such as s-ICAM1 and E-Selectin, which stops the rolling of leukocytes [45] (Figure 8).

Remarkably, one of the most important inhibitors of Th17 cell differentiation and development is IL-27, which showed reduced levels in SS patients. Importantly, IL-27 has pronounced pro- and anti-inflammatory action and is produced by antigen-presenting cells. This interleukin positively regulates both innate and adaptive immune responses, by positively regulating T and B lymphocyte activity, and negatively modulates immune responses contributing to immunosuppression. Interestingly, IL-27 synergizes with IL-12 to promote IFN-γ production by CD4, CD8 T cells and NK cells [46,47,48]. IL-27 was also identified as an early initiator of Th1 differentiation and inhibits differentiation of Th17 cells [49,50,51]. In addition, IL-27 negatively regulates neutrophil recruitment at the site of infection and its downregulation has demonstrated to increase the levels of neutrophils [52].

In our results, we observed higher levels of IL-27 in ICU controls (consisting of patients with stroke). Although IL-27 has been shown to be elevated in sepsis [53], our apparently contradictory results could be explained by the fact that IL-27 has been shown to be overexpressed in rodent models of intracerebral hemorrhage [54].

Regarding IP-10, also known as C-X-C motif chemokine ligand 10 (CXCL10), is secreted by several cell types in response to IFN-γ by monocytes [55], and is involved in an innate and adaptive immune response by contributing to the chemoattraction of monocytes/macrophages, T cells, NK cells, and dendritic cells and promoting T cell adhesion to endothelial cells [56,57]. This chemokine is highly expressed in SS patients; it has been shown that this chemokine recruits immunosuppressive CXCR3 (the cognate receptor)-expressing CD4 C/CD8 C effector T cells and Tregs. Thus, CXCR3 C Tregs may inhibit adaptive immune responses (via effector T cells and NK cells) [58].

Regarding IL-12, it plays a key role in the innate immune response by the activation of macrophages and NK cells. Our results showed that pro-inflammatory IL-12 was overexpressed in SS patients. Interestingly, IL-12 also showed a positive correlation with anti-inflammatory IL-10, indicating that SIRS and CARS are both simultaneously active in septic patients, as some authors have previously proposed [59], due to IL-10 is an anti-inflammatory cytokine mainly produced by monocytes. It downregulates the expression of Th1 cytokines, MHC class II antigens, and co-stimulatory molecules of macrophages. It also enhances B cell survival and proliferation, and antibody production. Moreover, IL-10 predominantly inhibits pro-inflammatory responses (e.g., TNF-α and IL-1β) induced by lipopolysaccharide and other bacterial products [60]. Interestingly, IL-12 also showed a moderate correlation with S100A9, an important protein which plays a prominent role in the regulation of inflammatory processes and immune response, such as neutrophil chemotaxis and adhesion induction [61] through extracellularly amplifying TLR-mediated responses.

Regarding the immune function mediated by the endothelium, some proteins are involved with macrophages and transendothelial migration such as GM-CSF, which is responsible for stimulating stem cells to produce granulocytes such as neutrophils and monocytes, and chemokines such as MIP-1α and MIP-1β and MCP-1, involved in the infiltration of monocytes/macrophages through the endothelium (Figure 8). Interestingly, all these proteins were highly expressed in patients with SS, indicating that the transendothelial migration of macrophages and neutrophils could be overactivated in SS patients. Our results evaluating the NLR ratio of immune cell subsets showed neutrophiles is the most abundant subpopulation and suggest that inflammation may be further contributed by neutrophiles through the production of IL-1α and IL-18, and pro-inflammatory IL-6 in SS patients [34]. Nevertheless, transendothelial migration ultimately depends on the binding of neutrophils and monocytes to the endothelium through E-selectin and s-ICAM (Figure 8), which in our study showed a positive correlation and also correlated with pyroptosis-related cytokines (Figure 7 and Tables S1–S3). Our results indicate that septic patients have the highest levels of E-selectin, supporting the idea that the endothelium collaborates with the immune response against infection (Figure 8). Importantly, SS patients also showed high levels of s-ICAM1 which also correlated with poor prognosis, thereby confirming the endothelial damage.

One of the most significant clusters we found was formed by coagulation-related factors, endothelial mediators, pyroptosis-related cytokines and the inflammatory cytokine IL-6 (Figure 7). Interestingly, while E-selectin allows weak binding of leukocytes to the endothelium and, therefore, their rolling towards the site of infection, s-ICAM1 induces strong binding, keeping the leukocytes at the site of infection to achieve its function [62,63]. Likewise, P-selectin is rapidly expressed in response to thrombin, and thus, it is involved in thrombus formation and platelet aggregation [64]. The close relationship among the different immune mediators may be a consequence of the relationship between these cytokines and thrombin levels [65,66].

P-selectin has been proposed as a central mediator of platelet aggregation and thrombus formation in sepsis [67]. Moreover, S100A8 is derived from neutrophils and can also promote coagulation through a mechanism involving the activation of platelets [68,69], a process which is also facilitated by P-selectin expression in the surface of platelets (Figure 8). Importantly E-selectin and P-selectin showed a moderate positive correlation in our study. We found differences in P-selectin levels when comparing sepsis and SS to the control ICU group, indicating that septic and SS patients could present enhanced platelet aggregation. In agreement, E-selectin levels were found in the same cluster with DD, indicating that the endothelium plays an important role in coagulation in SS.

Regarding the adaptive immune response, IL-4 and IL-13 were also analyzed. IL-4 acts as an anti-inflammatory by blocking the synthesis of IL-1, TNF-α, IL-6 and MIP-1 proteins. Furthermore, it promotes the differentiation of Th2 lymphocytes and the proliferation and differentiation of B lymphocytes [70,71]. Interestingly, IL-13 is mainly produced by Th2, and also modulates the production of IL-1, TNF, IL-8 and MIP-1 proteins, stimulates the growth and differentiation of B cells, and inhibits Th1 cells [72].

IL-4 was found in the same cluster together with GM-CSF and IFN-α. The overexpression of these proteins linked to MIP-1α (also called CCL3) and IFN-α suggests macrophage activation. IL-4 is produced by activated Th2 cells and together with Th17-derived IL-17A provide a chronic inflammatory milieu in critically ill patients. Interestingly, IL-4 can act as an anti-inflammatory molecule by blocking the synthesis of IL-1α, TNF-α, IL-6 and MIP-1 proteins [73] (Figure 8). Similarly, IL-13 is produced by T lymphocytes and plays a fundamental role in regulating the function of monocytes and B cells by modulating the production of IL-1α, IL-1β, TNF-α, IL-8 and MIP-1 proteins [74]. Furthermore, IL-4 stimulates the growth and differentiation of B cells, and inhibits Th1 cells and the production of inflammatory cytokines [75]. Our results showed that patients with SS did not show differences in IL-4 and IL-13 when compared to the other groups of patients, suggesting these cytokines may not inhibit the expression of IL-1α, IL-1β, IL-6 and T lymphocytes may not be completely activated in this group of patients (Figure 8). However, no differences in IL-4 and Il-13 could reflect a time point difference or differences in their removal in those patients. Therefore, as previously described by Gentile et al. and Hotchkins et al., the adaptive immune system cannot yet be fully activated, neither in septic nor in SS patients. In addition, immunosuppressive responses and pro-coagulation signals may be active in patients with SS [14,76]. Although one of the strengths of this work is that the patients enrolled were classified according the most recent consensus (SEPSIS-3) criteria, our work has the limitation of the low number of patients included in each group. In addition, it is noteworthy that some of these mediators, including pyroptosis-related cytokines, can also be produced by non-immune cells such as endothelial cells.

## 5. Conclusions

In this study we characterized the expression of several cytokines and immune mediators participating in the early and late immune response during the first stages of sepsis development. The highest levels of IL-6 and pyroptosis-related cytokines (IL-18 and IL-1α) were found in SS patients, which correlated with D-dimer. Moreover, endothelial function may be affected as shown by the overexpression of adhesion molecules such as s-ICAM1 and E-Selectin during septic shock.

## 6. Patents

JLG-G and FVP are inventors of a patent (EP3535587B1) related with the detection of circulating histones by mass spectrometry. Other authors declare no conflict of interest.

## Figures and Tables

**Table 1:** Clinical data in each subgroup: demographics, severity scores, analytical data and documented microbiologic etiology of the infectious process from the control intensive care unit, sepsis and septic shock patients.

Participant Characteristics	Control ICU Non-Septic Patients (*n* = 5)	Sepsis (*n* = 10)	Septic Shock (*n* = 15)	*p* ≤ 0.05
Age (years)	68 ± 8	68 ± 11	65 ± 15	n.s.
Male–female ratio	3:2	6:4	11:4	NA
APACHE II score	15 ± 4	18 ± 7	23 ± 7	n.s.
SOFA score 1st day	5 ± 3	6 ± 2	9 ± 3	0.004
CRP (mg/L)	8.2 ± 8.7	225.3 ± 153.7	277.1 ± 130.5	0.003
Procalcitonin (ng/mL)	0.5 ± 0.8	7.4 ± 9.7	41.2 ± 32.7	0.005
Lactate 1st h (mmol/L)	1.9 ± 0.3	1.9 ± 1.2	5.9 ± 4.7	0.009
Origin of infection	NA	Gram-positive = 22% Gram-negative = 11% Virus = 0% Others = 0% Not available: 67%	Gram-positive = 40% Gram-negative = 27% Virus = 6% Others = 0% Not available: 27%	NA
Body Mass Index	26.2 ± 2.6	28.6 ± 3.9	26.1 ± 2.4	n.s
ICU LOS (days)	4 ± 2	10 ± 13	7 ± 6	n.s
Hospital LOS (days)	14 ± 9	18 ± 11	13 ± 11	n.s
White blood cells	11,056 ± 4288	14,751 ± 12,106	14,887 ± 12,042	n.s
Glucose (mg/dL)	149 ± 26	164 ± 58	158 ± 60	n.s
Platelet’s count	279,600 ± 103,919	221,500 ± 176,025	154866 ± 91357	n.s
Antimicrobial first hour (%)	-	4 (40%)	11 (73%)	0.006
Vasopressor therapy (%)	1 (20%)	2 (20%)	14 (93%)	0.001
Renal Replacement Therapy (%)	-	-	4 (27%)	n.s
Mechanical Ventilation (%)	2 (40%)	-	3 (20%)	n.s
D-Dimer	NA	1377 ± 481	5512.75 ± 8045.76	0.045
Fibrin	NA	7.48 ± 2.34	5.87 ± 1.71	n.s
Direct WBC count	12,421.67 ± 5089.37	13,566.67 ± 11,868.66	14,730.20 ± 12,216.10	n.s

Note: ICU: intensive care unit; NA: not applicable; ns: non-significant; CRP: c-reactive protein; APACHE II: Acute Physiology and Chronic Health disease Classification System II; SOFA: Sequential [Sepsis-related] Organ Failure Assessment. Direct WBC count was assessed by means flow cytometry in the central laboratory of the Hospital Clínico Universitario de Valencia. Origin of infection refers to the entire stay of ICU. Values are expressed as median ± standard deviation.

## Data Availability

All results are showed in the manuscript or in the Supplementary Materials.

## Supplementary Materials

The following supporting information can be downloaded at: https://www.mdpi.com/article/10.3390/biomedicines10030525/s1, Figure S1: Immune cell population and platelets in critically ill, septic and septic shock patients; Figure S2: Graphical scheme of early immune response in septic shock patients showing cytokines and mediators released from different immune cells and endothelium analyzed in the present study; Table S1: Spearman’s rank correlation coefficients obtained analyzing different cytokines, immune mediators and clinical parameters; Table S2: *p*-value obtained using Spearman’s correlations analyzing different cytokines, immune mediators and clinical parameters; Table S3: Adj. *p*-value obtained using Spearman’s correlations analyzing different cytokines, immune mediators and clinical parameters.
