# Supplementary material for: Characterization of Early Peripheral Immune Responses in Patients with Sepsis and Septic Shock
Source: Biomedicines. 2022 Feb 23;10(3):525. doi: 10.3390/biomedicines10030525 (PMC8945007; doi:10.3390/biomedicines10030525)
Supplement: Supplementary file 1 [file biomedicines-10-00525-s001.zip › biomedicines-1525080-supplementary proof done/Supplementary material_Biomedicines_v3/Suppl. Figure S1.pptx]

## Slide 1
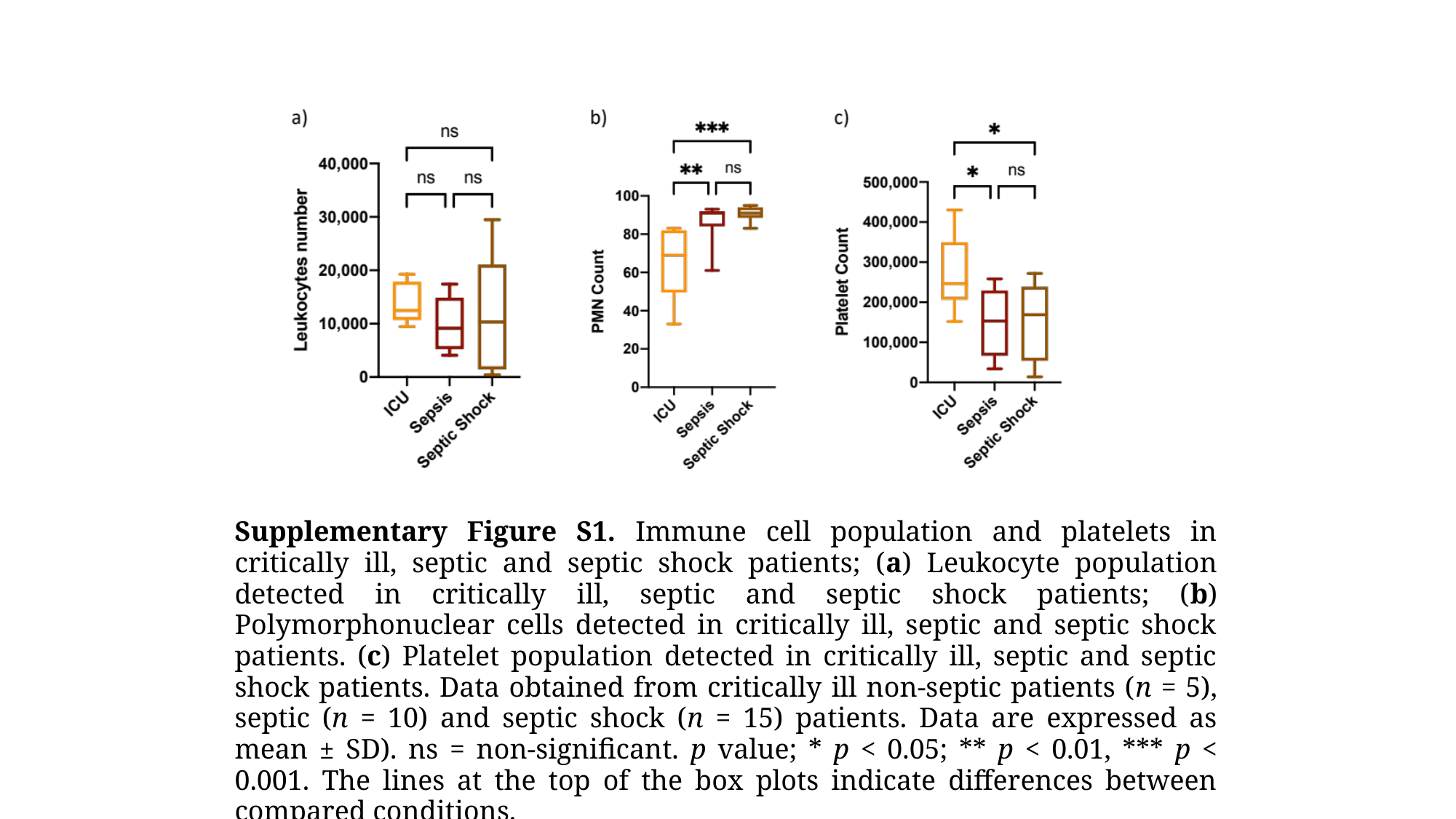

Supplementary Figure S1. Immune cell population and platelets in critically ill, septic and septic shock patients; (a) Leukocyte population detected in critically ill, septic and septic shock patients; (b) Polymorphonuclear cells detected in critically ill, septic and septic shock patients. (c) Platelet population detected in critically ill, septic and septic shock patients. Data obtained from critically ill non-septic patients (n = 5), septic (n = 10) and septic shock (n = 15) patients. Data are expressed as mean ± SD). ns = non-significant. p value; * p < 0.05; ** p < 0.01, *** p < 0.001. The lines at the top of the box plots indicate differences between compared conditions.
