# Supplementary material for: Characterization of Early Peripheral Immune Responses in Patients with Sepsis and Septic Shock
Source: Biomedicines. 2022 Feb 23;10(3):525. doi: 10.3390/biomedicines10030525 (PMC8945007; doi:10.3390/biomedicines10030525)
Supplement: Supplementary file 1 [file biomedicines-10-00525-s001.zip › biomedicines-1525080-supplementary proof done/Supplementary material_Biomedicines_v3/Supplementary figure S2_Biomedicines v2.pptx]

## Slide 1
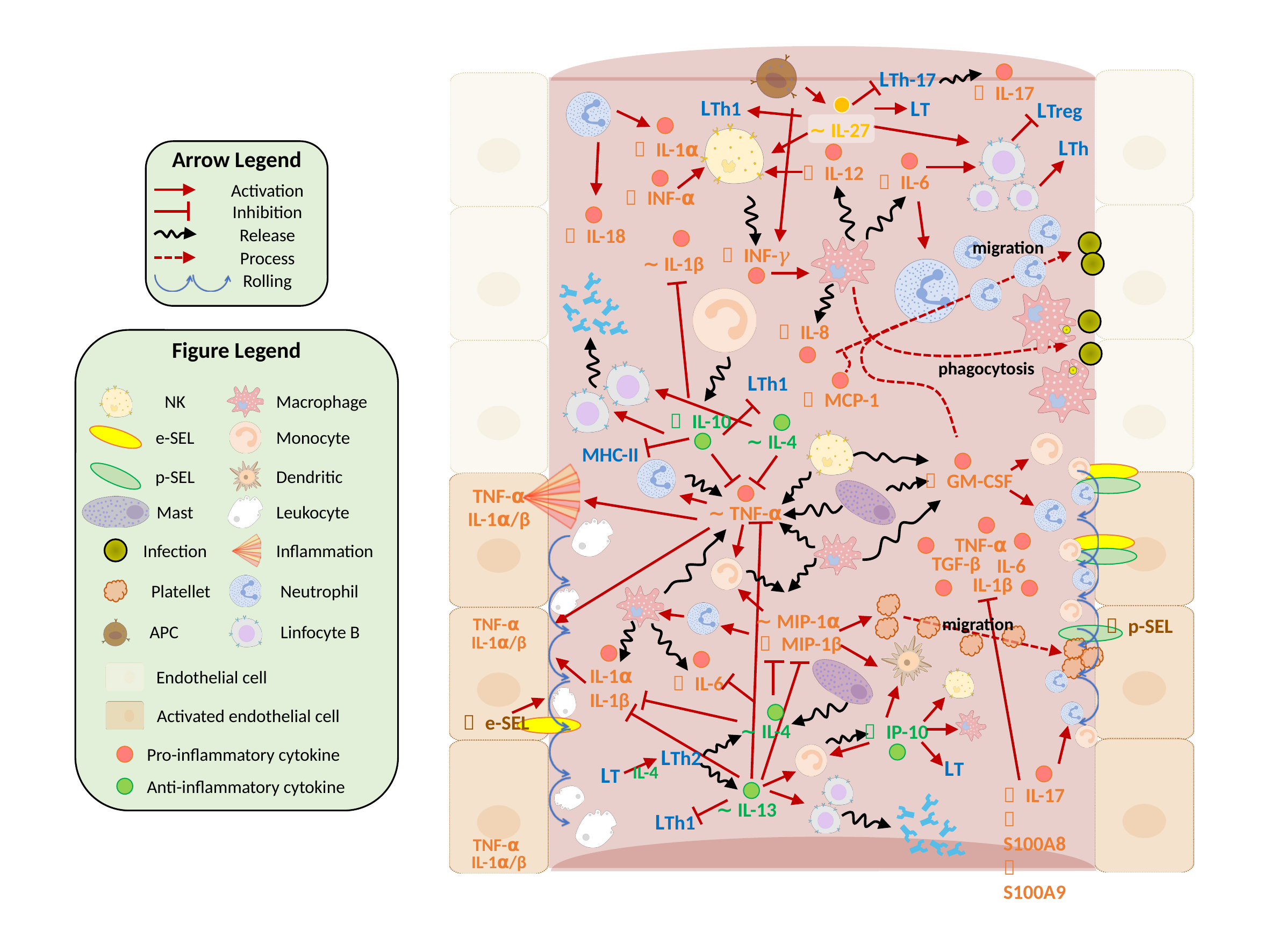

LTh-17
￪ IL-17
LTh1
LT
LTreg
∼ IL-27
￪ IL-1⍺
LTh
Arrow Legend
Activation
Inhibition
Release
Process
Rolling
Figure Legend
NK
Macrophage
e-SEL
Monocyte
p-SEL
Dendritic
Mast
Leukocyte
Infection
Inflammation
Platellet
Neutrophil
APC
Linfocyte B
Endothelial cell
Activated endothelial cell
Pro-inflammatory cytokine
Anti-inflammatory cytokine
￪ IL-12
￪ IL-6
￪ INF-⍺
￪ IL-18
∼ IL-1β
migration
￪ INF-𝛾
￪ IL-8
phagocytosis
LTh1
￪ MCP-1
￪ IL-10
∼ IL-4
MHC-II
￪ GM-CSF
TNF-⍺
∼ TNF-⍺
IL-1⍺/β
 TNF-⍺
 TGF-β
IL-6
IL-1β
∼ MIP-1⍺
migration
TNF-⍺
￪ p-SEL
￪ MIP-1β
IL-1⍺/β
IL-1⍺
IL-1β
￪ IL-6
∼ IL-4
￪ e-SEL
￪ IP-10
LTh2
LT
LT
 IL-4
￪ IL-17
￪ S100A8
￪ S100A9
∼ IL-13
LTh1
TNF-⍺
IL-1⍺/β
