# Supplementary material for: Characterization of Early Peripheral Immune Responses in Patients with Sepsis and Septic Shock
Source: Biomedicines. 2022 Feb 23;10(3):525. doi: 10.3390/biomedicines10030525 (PMC8945007; doi:10.3390/biomedicines10030525)
Supplement: Supplementary file 1 [file biomedicines-10-00525-s001.zip › biomedicines-1525080-supplementary proof done/Supplementary material_Biomedicines_v3/Supplementary Tables_Biomedicines_ v3 (2).pptx]

## Slide 1
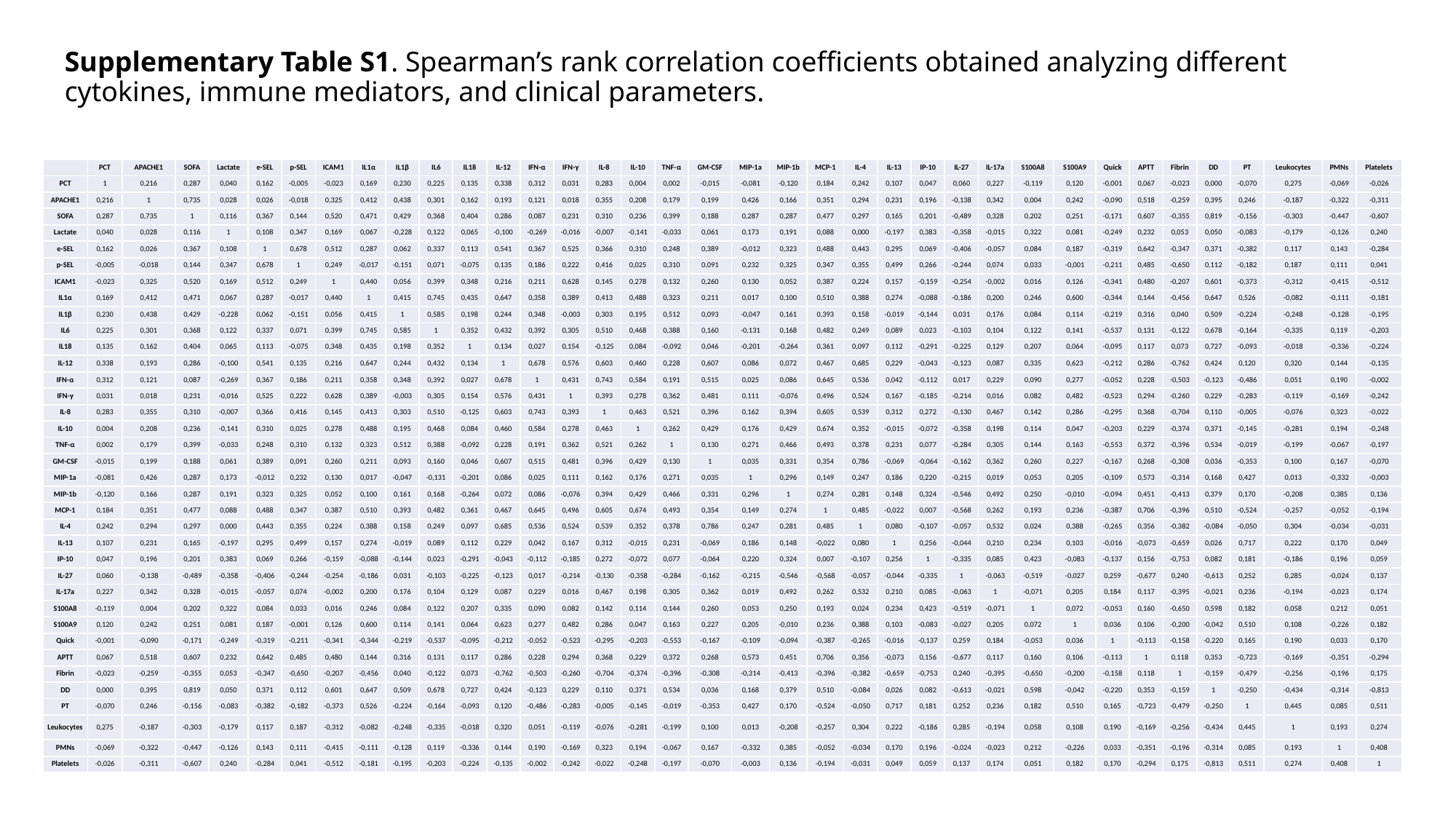

# Supplementary Table S1. Spearman’s rank correlation coefficients obtained analyzing different cytokines, immune mediators, and clinical parameters.
| | PCT | APACHE1 | SOFA | Lactate | e-SEL | p-SEL | ICAM1 | IL1α | IL1β | IL6 | IL18 | IL-12 | IFN-α | IFN-γ | IL-8 | IL-10 | TNF-α | GM-CSF | MIP-1a | MIP-1b | MCP-1 | IL-4 | IL-13 | IP-10 | IL-27 | IL-17a | S100A8 | S100A9 | Quick | APTT | Fibrin | DD | PT | Leukocytes | PMNs | Platelets |
| --- | --- | --- | --- | --- | --- | --- | --- | --- | --- | --- | --- | --- | --- | --- | --- | --- | --- | --- | --- | --- | --- | --- | --- | --- | --- | --- | --- | --- | --- | --- | --- | --- | --- | --- | --- | --- |
| PCT | 1 | 0,216 | 0,287 | 0,040 | 0,162 | -0,005 | -0,023 | 0,169 | 0,230 | 0,225 | 0,135 | 0,338 | 0,312 | 0,031 | 0,283 | 0,004 | 0,002 | -0,015 | -0,081 | -0,120 | 0,184 | 0,242 | 0,107 | 0,047 | 0,060 | 0,227 | -0,119 | 0,120 | -0,001 | 0,067 | -0,023 | 0,000 | -0,070 | 0,275 | -0,069 | -0,026 |
| APACHE1 | 0,216 | 1 | 0,735 | 0,028 | 0,026 | -0,018 | 0,325 | 0,412 | 0,438 | 0,301 | 0,162 | 0,193 | 0,121 | 0,018 | 0,355 | 0,208 | 0,179 | 0,199 | 0,426 | 0,166 | 0,351 | 0,294 | 0,231 | 0,196 | -0,138 | 0,342 | 0,004 | 0,242 | -0,090 | 0,518 | -0,259 | 0,395 | 0,246 | -0,187 | -0,322 | -0,311 |
| SOFA | 0,287 | 0,735 | 1 | 0,116 | 0,367 | 0,144 | 0,520 | 0,471 | 0,429 | 0,368 | 0,404 | 0,286 | 0,087 | 0,231 | 0,310 | 0,236 | 0,399 | 0,188 | 0,287 | 0,287 | 0,477 | 0,297 | 0,165 | 0,201 | -0,489 | 0,328 | 0,202 | 0,251 | -0,171 | 0,607 | -0,355 | 0,819 | -0,156 | -0,303 | -0,447 | -0,607 |
| Lactate | 0,040 | 0,028 | 0,116 | 1 | 0,108 | 0,347 | 0,169 | 0,067 | -0,228 | 0,122 | 0,065 | -0,100 | -0,269 | -0,016 | -0,007 | -0,141 | -0,033 | 0,061 | 0,173 | 0,191 | 0,088 | 0,000 | -0,197 | 0,383 | -0,358 | -0,015 | 0,322 | 0,081 | -0,249 | 0,232 | 0,053 | 0,050 | -0,083 | -0,179 | -0,126 | 0,240 |
| e-SEL | 0,162 | 0,026 | 0,367 | 0,108 | 1 | 0,678 | 0,512 | 0,287 | 0,062 | 0,337 | 0,113 | 0,541 | 0,367 | 0,525 | 0,366 | 0,310 | 0,248 | 0,389 | -0,012 | 0,323 | 0,488 | 0,443 | 0,295 | 0,069 | -0,406 | -0,057 | 0,084 | 0,187 | -0,319 | 0,642 | -0,347 | 0,371 | -0,382 | 0,117 | 0,143 | -0,284 |
| p-SEL | -0,005 | -0,018 | 0,144 | 0,347 | 0,678 | 1 | 0,249 | -0,017 | -0,151 | 0,071 | -0,075 | 0,135 | 0,186 | 0,222 | 0,416 | 0,025 | 0,310 | 0,091 | 0,232 | 0,325 | 0,347 | 0,355 | 0,499 | 0,266 | -0,244 | 0,074 | 0,033 | -0,001 | -0,211 | 0,485 | -0,650 | 0,112 | -0,182 | 0,187 | 0,111 | 0,041 |
| ICAM1 | -0,023 | 0,325 | 0,520 | 0,169 | 0,512 | 0,249 | 1 | 0,440 | 0,056 | 0,399 | 0,348 | 0,216 | 0,211 | 0,628 | 0,145 | 0,278 | 0,132 | 0,260 | 0,130 | 0,052 | 0,387 | 0,224 | 0,157 | -0,159 | -0,254 | -0,002 | 0,016 | 0,126 | -0,341 | 0,480 | -0,207 | 0,601 | -0,373 | -0,312 | -0,415 | -0,512 |
| IL1α | 0,169 | 0,412 | 0,471 | 0,067 | 0,287 | -0,017 | 0,440 | 1 | 0,415 | 0,745 | 0,435 | 0,647 | 0,358 | 0,389 | 0,413 | 0,488 | 0,323 | 0,211 | 0,017 | 0,100 | 0,510 | 0,388 | 0,274 | -0,088 | -0,186 | 0,200 | 0,246 | 0,600 | -0,344 | 0,144 | -0,456 | 0,647 | 0,526 | -0,082 | -0,111 | -0,181 |
| IL1β | 0,230 | 0,438 | 0,429 | -0,228 | 0,062 | -0,151 | 0,056 | 0,415 | 1 | 0,585 | 0,198 | 0,244 | 0,348 | -0,003 | 0,303 | 0,195 | 0,512 | 0,093 | -0,047 | 0,161 | 0,393 | 0,158 | -0,019 | -0,144 | 0,031 | 0,176 | 0,084 | 0,114 | -0,219 | 0,316 | 0,040 | 0,509 | -0,224 | -0,248 | -0,128 | -0,195 |
| IL6 | 0,225 | 0,301 | 0,368 | 0,122 | 0,337 | 0,071 | 0,399 | 0,745 | 0,585 | 1 | 0,352 | 0,432 | 0,392 | 0,305 | 0,510 | 0,468 | 0,388 | 0,160 | -0,131 | 0,168 | 0,482 | 0,249 | 0,089 | 0,023 | -0,103 | 0,104 | 0,122 | 0,141 | -0,537 | 0,131 | -0,122 | 0,678 | -0,164 | -0,335 | 0,119 | -0,203 |
| IL18 | 0,135 | 0,162 | 0,404 | 0,065 | 0,113 | -0,075 | 0,348 | 0,435 | 0,198 | 0,352 | 1 | 0,134 | 0,027 | 0,154 | -0,125 | 0,084 | -0,092 | 0,046 | -0,201 | -0,264 | 0,361 | 0,097 | 0,112 | -0,291 | -0,225 | 0,129 | 0,207 | 0,064 | -0,095 | 0,117 | 0,073 | 0,727 | -0,093 | -0,018 | -0,336 | -0,224 |
| IL-12 | 0,338 | 0,193 | 0,286 | -0,100 | 0,541 | 0,135 | 0,216 | 0,647 | 0,244 | 0,432 | 0,134 | 1 | 0,678 | 0,576 | 0,603 | 0,460 | 0,228 | 0,607 | 0,086 | 0,072 | 0,467 | 0,685 | 0,229 | -0,043 | -0,123 | 0,087 | 0,335 | 0,623 | -0,212 | 0,286 | -0,762 | 0,424 | 0,120 | 0,320 | 0,144 | -0,135 |
| IFN-α | 0,312 | 0,121 | 0,087 | -0,269 | 0,367 | 0,186 | 0,211 | 0,358 | 0,348 | 0,392 | 0,027 | 0,678 | 1 | 0,431 | 0,743 | 0,584 | 0,191 | 0,515 | 0,025 | 0,086 | 0,645 | 0,536 | 0,042 | -0,112 | 0,017 | 0,229 | 0,090 | 0,277 | -0,052 | 0,228 | -0,503 | -0,123 | -0,486 | 0,051 | 0,190 | -0,002 |
| IFN-γ | 0,031 | 0,018 | 0,231 | -0,016 | 0,525 | 0,222 | 0,628 | 0,389 | -0,003 | 0,305 | 0,154 | 0,576 | 0,431 | 1 | 0,393 | 0,278 | 0,362 | 0,481 | 0,111 | -0,076 | 0,496 | 0,524 | 0,167 | -0,185 | -0,214 | 0,016 | 0,082 | 0,482 | -0,523 | 0,294 | -0,260 | 0,229 | -0,283 | -0,119 | -0,169 | -0,242 |
| IL-8 | 0,283 | 0,355 | 0,310 | -0,007 | 0,366 | 0,416 | 0,145 | 0,413 | 0,303 | 0,510 | -0,125 | 0,603 | 0,743 | 0,393 | 1 | 0,463 | 0,521 | 0,396 | 0,162 | 0,394 | 0,605 | 0,539 | 0,312 | 0,272 | -0,130 | 0,467 | 0,142 | 0,286 | -0,295 | 0,368 | -0,704 | 0,110 | -0,005 | -0,076 | 0,323 | -0,022 |
| IL-10 | 0,004 | 0,208 | 0,236 | -0,141 | 0,310 | 0,025 | 0,278 | 0,488 | 0,195 | 0,468 | 0,084 | 0,460 | 0,584 | 0,278 | 0,463 | 1 | 0,262 | 0,429 | 0,176 | 0,429 | 0,674 | 0,352 | -0,015 | -0,072 | -0,358 | 0,198 | 0,114 | 0,047 | -0,203 | 0,229 | -0,374 | 0,371 | -0,145 | -0,281 | 0,194 | -0,248 |
| TNF-α | 0,002 | 0,179 | 0,399 | -0,033 | 0,248 | 0,310 | 0,132 | 0,323 | 0,512 | 0,388 | -0,092 | 0,228 | 0,191 | 0,362 | 0,521 | 0,262 | 1 | 0,130 | 0,271 | 0,466 | 0,493 | 0,378 | 0,231 | 0,077 | -0,284 | 0,305 | 0,144 | 0,163 | -0,553 | 0,372 | -0,396 | 0,534 | -0,019 | -0,199 | -0,067 | -0,197 |
| GM-CSF | -0,015 | 0,199 | 0,188 | 0,061 | 0,389 | 0,091 | 0,260 | 0,211 | 0,093 | 0,160 | 0,046 | 0,607 | 0,515 | 0,481 | 0,396 | 0,429 | 0,130 | 1 | 0,035 | 0,331 | 0,354 | 0,786 | -0,069 | -0,064 | -0,162 | 0,362 | 0,260 | 0,227 | -0,167 | 0,268 | -0,308 | 0,036 | -0,353 | 0,100 | 0,167 | -0,070 |
| MIP-1a | -0,081 | 0,426 | 0,287 | 0,173 | -0,012 | 0,232 | 0,130 | 0,017 | -0,047 | -0,131 | -0,201 | 0,086 | 0,025 | 0,111 | 0,162 | 0,176 | 0,271 | 0,035 | 1 | 0,296 | 0,149 | 0,247 | 0,186 | 0,220 | -0,215 | 0,019 | 0,053 | 0,205 | -0,109 | 0,573 | -0,314 | 0,168 | 0,427 | 0,013 | -0,332 | -0,003 |
| MIP-1b | -0,120 | 0,166 | 0,287 | 0,191 | 0,323 | 0,325 | 0,052 | 0,100 | 0,161 | 0,168 | -0,264 | 0,072 | 0,086 | -0,076 | 0,394 | 0,429 | 0,466 | 0,331 | 0,296 | 1 | 0,274 | 0,281 | 0,148 | 0,324 | -0,546 | 0,492 | 0,250 | -0,010 | -0,094 | 0,451 | -0,413 | 0,379 | 0,170 | -0,208 | 0,385 | 0,136 |
| MCP-1 | 0,184 | 0,351 | 0,477 | 0,088 | 0,488 | 0,347 | 0,387 | 0,510 | 0,393 | 0,482 | 0,361 | 0,467 | 0,645 | 0,496 | 0,605 | 0,674 | 0,493 | 0,354 | 0,149 | 0,274 | 1 | 0,485 | -0,022 | 0,007 | -0,568 | 0,262 | 0,193 | 0,236 | -0,387 | 0,706 | -0,396 | 0,510 | -0,524 | -0,257 | -0,052 | -0,194 |
| IL-4 | 0,242 | 0,294 | 0,297 | 0,000 | 0,443 | 0,355 | 0,224 | 0,388 | 0,158 | 0,249 | 0,097 | 0,685 | 0,536 | 0,524 | 0,539 | 0,352 | 0,378 | 0,786 | 0,247 | 0,281 | 0,485 | 1 | 0,080 | -0,107 | -0,057 | 0,532 | 0,024 | 0,388 | -0,265 | 0,356 | -0,382 | -0,084 | -0,050 | 0,304 | -0,034 | -0,031 |
| IL-13 | 0,107 | 0,231 | 0,165 | -0,197 | 0,295 | 0,499 | 0,157 | 0,274 | -0,019 | 0,089 | 0,112 | 0,229 | 0,042 | 0,167 | 0,312 | -0,015 | 0,231 | -0,069 | 0,186 | 0,148 | -0,022 | 0,080 | 1 | 0,256 | -0,044 | 0,210 | 0,234 | 0,103 | -0,016 | -0,073 | -0,659 | 0,026 | 0,717 | 0,222 | 0,170 | 0,049 |
| IP-10 | 0,047 | 0,196 | 0,201 | 0,383 | 0,069 | 0,266 | -0,159 | -0,088 | -0,144 | 0,023 | -0,291 | -0,043 | -0,112 | -0,185 | 0,272 | -0,072 | 0,077 | -0,064 | 0,220 | 0,324 | 0,007 | -0,107 | 0,256 | 1 | -0,335 | 0,085 | 0,423 | -0,083 | -0,137 | 0,156 | -0,753 | 0,082 | 0,181 | -0,186 | 0,196 | 0,059 |
| IL-27 | 0,060 | -0,138 | -0,489 | -0,358 | -0,406 | -0,244 | -0,254 | -0,186 | 0,031 | -0,103 | -0,225 | -0,123 | 0,017 | -0,214 | -0,130 | -0,358 | -0,284 | -0,162 | -0,215 | -0,546 | -0,568 | -0,057 | -0,044 | -0,335 | 1 | -0,063 | -0,519 | -0,027 | 0,259 | -0,677 | 0,240 | -0,613 | 0,252 | 0,285 | -0,024 | 0,137 |
| IL-17a | 0,227 | 0,342 | 0,328 | -0,015 | -0,057 | 0,074 | -0,002 | 0,200 | 0,176 | 0,104 | 0,129 | 0,087 | 0,229 | 0,016 | 0,467 | 0,198 | 0,305 | 0,362 | 0,019 | 0,492 | 0,262 | 0,532 | 0,210 | 0,085 | -0,063 | 1 | -0,071 | 0,205 | 0,184 | 0,117 | -0,395 | -0,021 | 0,236 | -0,194 | -0,023 | 0,174 |
| S100A8 | -0,119 | 0,004 | 0,202 | 0,322 | 0,084 | 0,033 | 0,016 | 0,246 | 0,084 | 0,122 | 0,207 | 0,335 | 0,090 | 0,082 | 0,142 | 0,114 | 0,144 | 0,260 | 0,053 | 0,250 | 0,193 | 0,024 | 0,234 | 0,423 | -0,519 | -0,071 | 1 | 0,072 | -0,053 | 0,160 | -0,650 | 0,598 | 0,182 | 0,058 | 0,212 | 0,051 |
| S100A9 | 0,120 | 0,242 | 0,251 | 0,081 | 0,187 | -0,001 | 0,126 | 0,600 | 0,114 | 0,141 | 0,064 | 0,623 | 0,277 | 0,482 | 0,286 | 0,047 | 0,163 | 0,227 | 0,205 | -0,010 | 0,236 | 0,388 | 0,103 | -0,083 | -0,027 | 0,205 | 0,072 | 1 | 0,036 | 0,106 | -0,200 | -0,042 | 0,510 | 0,108 | -0,226 | 0,182 |
| Quick | -0,001 | -0,090 | -0,171 | -0,249 | -0,319 | -0,211 | -0,341 | -0,344 | -0,219 | -0,537 | -0,095 | -0,212 | -0,052 | -0,523 | -0,295 | -0,203 | -0,553 | -0,167 | -0,109 | -0,094 | -0,387 | -0,265 | -0,016 | -0,137 | 0,259 | 0,184 | -0,053 | 0,036 | 1 | -0,113 | -0,158 | -0,220 | 0,165 | 0,190 | 0,033 | 0,170 |
| APTT | 0,067 | 0,518 | 0,607 | 0,232 | 0,642 | 0,485 | 0,480 | 0,144 | 0,316 | 0,131 | 0,117 | 0,286 | 0,228 | 0,294 | 0,368 | 0,229 | 0,372 | 0,268 | 0,573 | 0,451 | 0,706 | 0,356 | -0,073 | 0,156 | -0,677 | 0,117 | 0,160 | 0,106 | -0,113 | 1 | 0,118 | 0,353 | -0,723 | -0,169 | -0,351 | -0,294 |
| Fibrin | -0,023 | -0,259 | -0,355 | 0,053 | -0,347 | -0,650 | -0,207 | -0,456 | 0,040 | -0,122 | 0,073 | -0,762 | -0,503 | -0,260 | -0,704 | -0,374 | -0,396 | -0,308 | -0,314 | -0,413 | -0,396 | -0,382 | -0,659 | -0,753 | 0,240 | -0,395 | -0,650 | -0,200 | -0,158 | 0,118 | 1 | -0,159 | -0,479 | -0,256 | -0,196 | 0,175 |
| DD | 0,000 | 0,395 | 0,819 | 0,050 | 0,371 | 0,112 | 0,601 | 0,647 | 0,509 | 0,678 | 0,727 | 0,424 | -0,123 | 0,229 | 0,110 | 0,371 | 0,534 | 0,036 | 0,168 | 0,379 | 0,510 | -0,084 | 0,026 | 0,082 | -0,613 | -0,021 | 0,598 | -0,042 | -0,220 | 0,353 | -0,159 | 1 | -0,250 | -0,434 | -0,314 | -0,813 |
| PT | -0,070 | 0,246 | -0,156 | -0,083 | -0,382 | -0,182 | -0,373 | 0,526 | -0,224 | -0,164 | -0,093 | 0,120 | -0,486 | -0,283 | -0,005 | -0,145 | -0,019 | -0,353 | 0,427 | 0,170 | -0,524 | -0,050 | 0,717 | 0,181 | 0,252 | 0,236 | 0,182 | 0,510 | 0,165 | -0,723 | -0,479 | -0,250 | 1 | 0,445 | 0,085 | 0,511 |
| Leukocytes | 0,275 | -0,187 | -0,303 | -0,179 | 0,117 | 0,187 | -0,312 | -0,082 | -0,248 | -0,335 | -0,018 | 0,320 | 0,051 | -0,119 | -0,076 | -0,281 | -0,199 | 0,100 | 0,013 | -0,208 | -0,257 | 0,304 | 0,222 | -0,186 | 0,285 | -0,194 | 0,058 | 0,108 | 0,190 | -0,169 | -0,256 | -0,434 | 0,445 | 1 | 0,193 | 0,274 |
| PMNs | -0,069 | -0,322 | -0,447 | -0,126 | 0,143 | 0,111 | -0,415 | -0,111 | -0,128 | 0,119 | -0,336 | 0,144 | 0,190 | -0,169 | 0,323 | 0,194 | -0,067 | 0,167 | -0,332 | 0,385 | -0,052 | -0,034 | 0,170 | 0,196 | -0,024 | -0,023 | 0,212 | -0,226 | 0,033 | -0,351 | -0,196 | -0,314 | 0,085 | 0,193 | 1 | 0,408 |
| Platelets | -0,026 | -0,311 | -0,607 | 0,240 | -0,284 | 0,041 | -0,512 | -0,181 | -0,195 | -0,203 | -0,224 | -0,135 | -0,002 | -0,242 | -0,022 | -0,248 | -0,197 | -0,070 | -0,003 | 0,136 | -0,194 | -0,031 | 0,049 | 0,059 | 0,137 | 0,174 | 0,051 | 0,182 | 0,170 | -0,294 | 0,175 | -0,813 | 0,511 | 0,274 | 0,408 | 1 |

## Slide 2
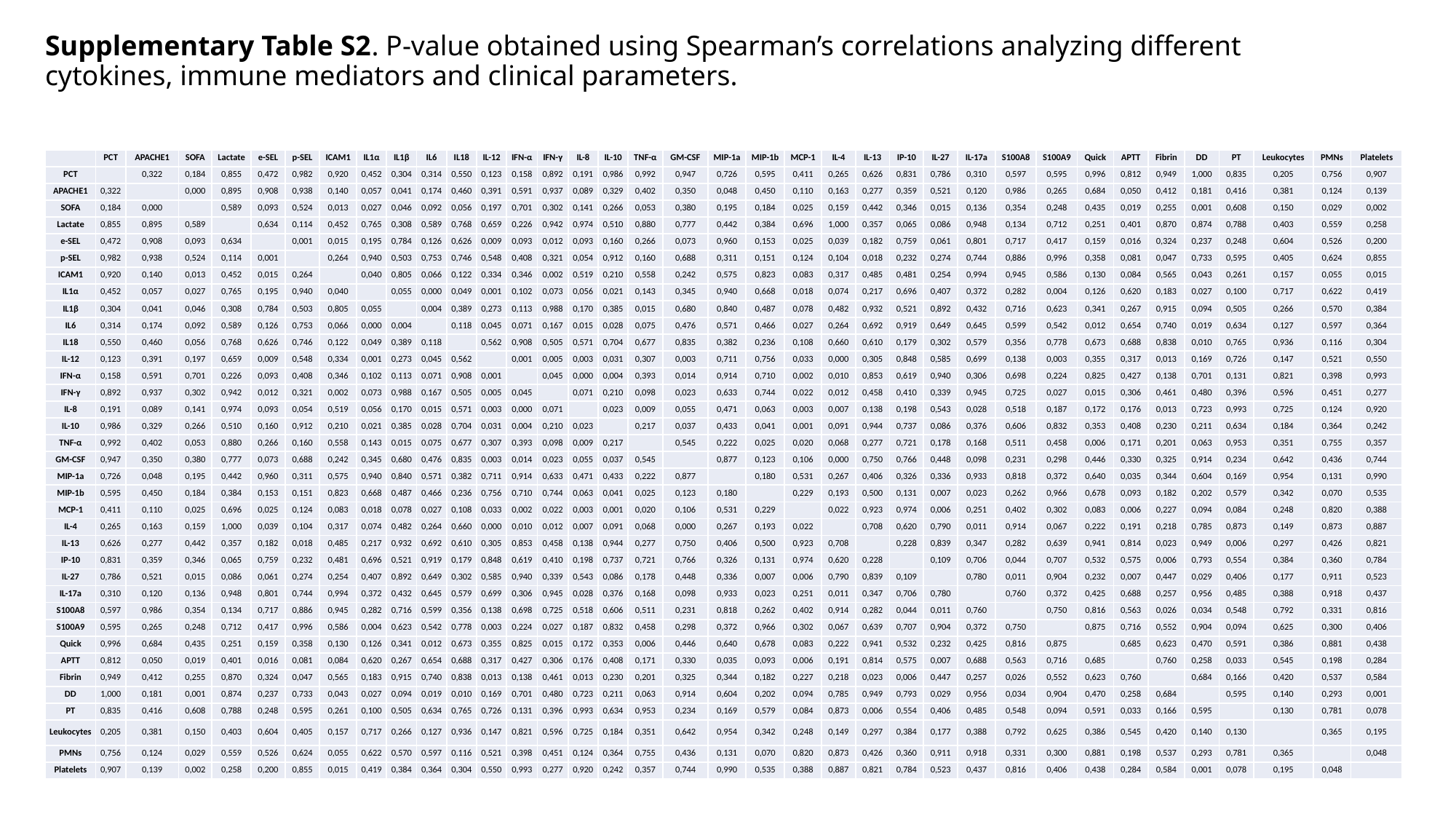

# Supplementary Table S2. P-value obtained using Spearman’s correlations analyzing different cytokines, immune mediators and clinical parameters.
| | PCT | APACHE1 | SOFA | Lactate | e-SEL | p-SEL | ICAM1 | IL1α | IL1β | IL6 | IL18 | IL-12 | IFN-α | IFN-γ | IL-8 | IL-10 | TNF-α | GM-CSF | MIP-1a | MIP-1b | MCP-1 | IL-4 | IL-13 | IP-10 | IL-27 | IL-17a | S100A8 | S100A9 | Quick | APTT | Fibrin | DD | PT | Leukocytes | PMNs | Platelets |
| --- | --- | --- | --- | --- | --- | --- | --- | --- | --- | --- | --- | --- | --- | --- | --- | --- | --- | --- | --- | --- | --- | --- | --- | --- | --- | --- | --- | --- | --- | --- | --- | --- | --- | --- | --- | --- |
| PCT | | 0,322 | 0,184 | 0,855 | 0,472 | 0,982 | 0,920 | 0,452 | 0,304 | 0,314 | 0,550 | 0,123 | 0,158 | 0,892 | 0,191 | 0,986 | 0,992 | 0,947 | 0,726 | 0,595 | 0,411 | 0,265 | 0,626 | 0,831 | 0,786 | 0,310 | 0,597 | 0,595 | 0,996 | 0,812 | 0,949 | 1,000 | 0,835 | 0,205 | 0,756 | 0,907 |
| APACHE1 | 0,322 | | 0,000 | 0,895 | 0,908 | 0,938 | 0,140 | 0,057 | 0,041 | 0,174 | 0,460 | 0,391 | 0,591 | 0,937 | 0,089 | 0,329 | 0,402 | 0,350 | 0,048 | 0,450 | 0,110 | 0,163 | 0,277 | 0,359 | 0,521 | 0,120 | 0,986 | 0,265 | 0,684 | 0,050 | 0,412 | 0,181 | 0,416 | 0,381 | 0,124 | 0,139 |
| SOFA | 0,184 | 0,000 | | 0,589 | 0,093 | 0,524 | 0,013 | 0,027 | 0,046 | 0,092 | 0,056 | 0,197 | 0,701 | 0,302 | 0,141 | 0,266 | 0,053 | 0,380 | 0,195 | 0,184 | 0,025 | 0,159 | 0,442 | 0,346 | 0,015 | 0,136 | 0,354 | 0,248 | 0,435 | 0,019 | 0,255 | 0,001 | 0,608 | 0,150 | 0,029 | 0,002 |
| Lactate | 0,855 | 0,895 | 0,589 | | 0,634 | 0,114 | 0,452 | 0,765 | 0,308 | 0,589 | 0,768 | 0,659 | 0,226 | 0,942 | 0,974 | 0,510 | 0,880 | 0,777 | 0,442 | 0,384 | 0,696 | 1,000 | 0,357 | 0,065 | 0,086 | 0,948 | 0,134 | 0,712 | 0,251 | 0,401 | 0,870 | 0,874 | 0,788 | 0,403 | 0,559 | 0,258 |
| e-SEL | 0,472 | 0,908 | 0,093 | 0,634 | | 0,001 | 0,015 | 0,195 | 0,784 | 0,126 | 0,626 | 0,009 | 0,093 | 0,012 | 0,093 | 0,160 | 0,266 | 0,073 | 0,960 | 0,153 | 0,025 | 0,039 | 0,182 | 0,759 | 0,061 | 0,801 | 0,717 | 0,417 | 0,159 | 0,016 | 0,324 | 0,237 | 0,248 | 0,604 | 0,526 | 0,200 |
| p-SEL | 0,982 | 0,938 | 0,524 | 0,114 | 0,001 | | 0,264 | 0,940 | 0,503 | 0,753 | 0,746 | 0,548 | 0,408 | 0,321 | 0,054 | 0,912 | 0,160 | 0,688 | 0,311 | 0,151 | 0,124 | 0,104 | 0,018 | 0,232 | 0,274 | 0,744 | 0,886 | 0,996 | 0,358 | 0,081 | 0,047 | 0,733 | 0,595 | 0,405 | 0,624 | 0,855 |
| ICAM1 | 0,920 | 0,140 | 0,013 | 0,452 | 0,015 | 0,264 | | 0,040 | 0,805 | 0,066 | 0,122 | 0,334 | 0,346 | 0,002 | 0,519 | 0,210 | 0,558 | 0,242 | 0,575 | 0,823 | 0,083 | 0,317 | 0,485 | 0,481 | 0,254 | 0,994 | 0,945 | 0,586 | 0,130 | 0,084 | 0,565 | 0,043 | 0,261 | 0,157 | 0,055 | 0,015 |
| IL1α | 0,452 | 0,057 | 0,027 | 0,765 | 0,195 | 0,940 | 0,040 | | 0,055 | 0,000 | 0,049 | 0,001 | 0,102 | 0,073 | 0,056 | 0,021 | 0,143 | 0,345 | 0,940 | 0,668 | 0,018 | 0,074 | 0,217 | 0,696 | 0,407 | 0,372 | 0,282 | 0,004 | 0,126 | 0,620 | 0,183 | 0,027 | 0,100 | 0,717 | 0,622 | 0,419 |
| IL1β | 0,304 | 0,041 | 0,046 | 0,308 | 0,784 | 0,503 | 0,805 | 0,055 | | 0,004 | 0,389 | 0,273 | 0,113 | 0,988 | 0,170 | 0,385 | 0,015 | 0,680 | 0,840 | 0,487 | 0,078 | 0,482 | 0,932 | 0,521 | 0,892 | 0,432 | 0,716 | 0,623 | 0,341 | 0,267 | 0,915 | 0,094 | 0,505 | 0,266 | 0,570 | 0,384 |
| IL6 | 0,314 | 0,174 | 0,092 | 0,589 | 0,126 | 0,753 | 0,066 | 0,000 | 0,004 | | 0,118 | 0,045 | 0,071 | 0,167 | 0,015 | 0,028 | 0,075 | 0,476 | 0,571 | 0,466 | 0,027 | 0,264 | 0,692 | 0,919 | 0,649 | 0,645 | 0,599 | 0,542 | 0,012 | 0,654 | 0,740 | 0,019 | 0,634 | 0,127 | 0,597 | 0,364 |
| IL18 | 0,550 | 0,460 | 0,056 | 0,768 | 0,626 | 0,746 | 0,122 | 0,049 | 0,389 | 0,118 | | 0,562 | 0,908 | 0,505 | 0,571 | 0,704 | 0,677 | 0,835 | 0,382 | 0,236 | 0,108 | 0,660 | 0,610 | 0,179 | 0,302 | 0,579 | 0,356 | 0,778 | 0,673 | 0,688 | 0,838 | 0,010 | 0,765 | 0,936 | 0,116 | 0,304 |
| IL-12 | 0,123 | 0,391 | 0,197 | 0,659 | 0,009 | 0,548 | 0,334 | 0,001 | 0,273 | 0,045 | 0,562 | | 0,001 | 0,005 | 0,003 | 0,031 | 0,307 | 0,003 | 0,711 | 0,756 | 0,033 | 0,000 | 0,305 | 0,848 | 0,585 | 0,699 | 0,138 | 0,003 | 0,355 | 0,317 | 0,013 | 0,169 | 0,726 | 0,147 | 0,521 | 0,550 |
| IFN-α | 0,158 | 0,591 | 0,701 | 0,226 | 0,093 | 0,408 | 0,346 | 0,102 | 0,113 | 0,071 | 0,908 | 0,001 | | 0,045 | 0,000 | 0,004 | 0,393 | 0,014 | 0,914 | 0,710 | 0,002 | 0,010 | 0,853 | 0,619 | 0,940 | 0,306 | 0,698 | 0,224 | 0,825 | 0,427 | 0,138 | 0,701 | 0,131 | 0,821 | 0,398 | 0,993 |
| IFN-γ | 0,892 | 0,937 | 0,302 | 0,942 | 0,012 | 0,321 | 0,002 | 0,073 | 0,988 | 0,167 | 0,505 | 0,005 | 0,045 | | 0,071 | 0,210 | 0,098 | 0,023 | 0,633 | 0,744 | 0,022 | 0,012 | 0,458 | 0,410 | 0,339 | 0,945 | 0,725 | 0,027 | 0,015 | 0,306 | 0,461 | 0,480 | 0,396 | 0,596 | 0,451 | 0,277 |
| IL-8 | 0,191 | 0,089 | 0,141 | 0,974 | 0,093 | 0,054 | 0,519 | 0,056 | 0,170 | 0,015 | 0,571 | 0,003 | 0,000 | 0,071 | | 0,023 | 0,009 | 0,055 | 0,471 | 0,063 | 0,003 | 0,007 | 0,138 | 0,198 | 0,543 | 0,028 | 0,518 | 0,187 | 0,172 | 0,176 | 0,013 | 0,723 | 0,993 | 0,725 | 0,124 | 0,920 |
| IL-10 | 0,986 | 0,329 | 0,266 | 0,510 | 0,160 | 0,912 | 0,210 | 0,021 | 0,385 | 0,028 | 0,704 | 0,031 | 0,004 | 0,210 | 0,023 | | 0,217 | 0,037 | 0,433 | 0,041 | 0,001 | 0,091 | 0,944 | 0,737 | 0,086 | 0,376 | 0,606 | 0,832 | 0,353 | 0,408 | 0,230 | 0,211 | 0,634 | 0,184 | 0,364 | 0,242 |
| TNF-α | 0,992 | 0,402 | 0,053 | 0,880 | 0,266 | 0,160 | 0,558 | 0,143 | 0,015 | 0,075 | 0,677 | 0,307 | 0,393 | 0,098 | 0,009 | 0,217 | | 0,545 | 0,222 | 0,025 | 0,020 | 0,068 | 0,277 | 0,721 | 0,178 | 0,168 | 0,511 | 0,458 | 0,006 | 0,171 | 0,201 | 0,063 | 0,953 | 0,351 | 0,755 | 0,357 |
| GM-CSF | 0,947 | 0,350 | 0,380 | 0,777 | 0,073 | 0,688 | 0,242 | 0,345 | 0,680 | 0,476 | 0,835 | 0,003 | 0,014 | 0,023 | 0,055 | 0,037 | 0,545 | | 0,877 | 0,123 | 0,106 | 0,000 | 0,750 | 0,766 | 0,448 | 0,098 | 0,231 | 0,298 | 0,446 | 0,330 | 0,325 | 0,914 | 0,234 | 0,642 | 0,436 | 0,744 |
| MIP-1a | 0,726 | 0,048 | 0,195 | 0,442 | 0,960 | 0,311 | 0,575 | 0,940 | 0,840 | 0,571 | 0,382 | 0,711 | 0,914 | 0,633 | 0,471 | 0,433 | 0,222 | 0,877 | | 0,180 | 0,531 | 0,267 | 0,406 | 0,326 | 0,336 | 0,933 | 0,818 | 0,372 | 0,640 | 0,035 | 0,344 | 0,604 | 0,169 | 0,954 | 0,131 | 0,990 |
| MIP-1b | 0,595 | 0,450 | 0,184 | 0,384 | 0,153 | 0,151 | 0,823 | 0,668 | 0,487 | 0,466 | 0,236 | 0,756 | 0,710 | 0,744 | 0,063 | 0,041 | 0,025 | 0,123 | 0,180 | | 0,229 | 0,193 | 0,500 | 0,131 | 0,007 | 0,023 | 0,262 | 0,966 | 0,678 | 0,093 | 0,182 | 0,202 | 0,579 | 0,342 | 0,070 | 0,535 |
| MCP-1 | 0,411 | 0,110 | 0,025 | 0,696 | 0,025 | 0,124 | 0,083 | 0,018 | 0,078 | 0,027 | 0,108 | 0,033 | 0,002 | 0,022 | 0,003 | 0,001 | 0,020 | 0,106 | 0,531 | 0,229 | | 0,022 | 0,923 | 0,974 | 0,006 | 0,251 | 0,402 | 0,302 | 0,083 | 0,006 | 0,227 | 0,094 | 0,084 | 0,248 | 0,820 | 0,388 |
| IL-4 | 0,265 | 0,163 | 0,159 | 1,000 | 0,039 | 0,104 | 0,317 | 0,074 | 0,482 | 0,264 | 0,660 | 0,000 | 0,010 | 0,012 | 0,007 | 0,091 | 0,068 | 0,000 | 0,267 | 0,193 | 0,022 | | 0,708 | 0,620 | 0,790 | 0,011 | 0,914 | 0,067 | 0,222 | 0,191 | 0,218 | 0,785 | 0,873 | 0,149 | 0,873 | 0,887 |
| IL-13 | 0,626 | 0,277 | 0,442 | 0,357 | 0,182 | 0,018 | 0,485 | 0,217 | 0,932 | 0,692 | 0,610 | 0,305 | 0,853 | 0,458 | 0,138 | 0,944 | 0,277 | 0,750 | 0,406 | 0,500 | 0,923 | 0,708 | | 0,228 | 0,839 | 0,347 | 0,282 | 0,639 | 0,941 | 0,814 | 0,023 | 0,949 | 0,006 | 0,297 | 0,426 | 0,821 |
| IP-10 | 0,831 | 0,359 | 0,346 | 0,065 | 0,759 | 0,232 | 0,481 | 0,696 | 0,521 | 0,919 | 0,179 | 0,848 | 0,619 | 0,410 | 0,198 | 0,737 | 0,721 | 0,766 | 0,326 | 0,131 | 0,974 | 0,620 | 0,228 | | 0,109 | 0,706 | 0,044 | 0,707 | 0,532 | 0,575 | 0,006 | 0,793 | 0,554 | 0,384 | 0,360 | 0,784 |
| IL-27 | 0,786 | 0,521 | 0,015 | 0,086 | 0,061 | 0,274 | 0,254 | 0,407 | 0,892 | 0,649 | 0,302 | 0,585 | 0,940 | 0,339 | 0,543 | 0,086 | 0,178 | 0,448 | 0,336 | 0,007 | 0,006 | 0,790 | 0,839 | 0,109 | | 0,780 | 0,011 | 0,904 | 0,232 | 0,007 | 0,447 | 0,029 | 0,406 | 0,177 | 0,911 | 0,523 |
| IL-17a | 0,310 | 0,120 | 0,136 | 0,948 | 0,801 | 0,744 | 0,994 | 0,372 | 0,432 | 0,645 | 0,579 | 0,699 | 0,306 | 0,945 | 0,028 | 0,376 | 0,168 | 0,098 | 0,933 | 0,023 | 0,251 | 0,011 | 0,347 | 0,706 | 0,780 | | 0,760 | 0,372 | 0,425 | 0,688 | 0,257 | 0,956 | 0,485 | 0,388 | 0,918 | 0,437 |
| S100A8 | 0,597 | 0,986 | 0,354 | 0,134 | 0,717 | 0,886 | 0,945 | 0,282 | 0,716 | 0,599 | 0,356 | 0,138 | 0,698 | 0,725 | 0,518 | 0,606 | 0,511 | 0,231 | 0,818 | 0,262 | 0,402 | 0,914 | 0,282 | 0,044 | 0,011 | 0,760 | | 0,750 | 0,816 | 0,563 | 0,026 | 0,034 | 0,548 | 0,792 | 0,331 | 0,816 |
| S100A9 | 0,595 | 0,265 | 0,248 | 0,712 | 0,417 | 0,996 | 0,586 | 0,004 | 0,623 | 0,542 | 0,778 | 0,003 | 0,224 | 0,027 | 0,187 | 0,832 | 0,458 | 0,298 | 0,372 | 0,966 | 0,302 | 0,067 | 0,639 | 0,707 | 0,904 | 0,372 | 0,750 | | 0,875 | 0,716 | 0,552 | 0,904 | 0,094 | 0,625 | 0,300 | 0,406 |
| Quick | 0,996 | 0,684 | 0,435 | 0,251 | 0,159 | 0,358 | 0,130 | 0,126 | 0,341 | 0,012 | 0,673 | 0,355 | 0,825 | 0,015 | 0,172 | 0,353 | 0,006 | 0,446 | 0,640 | 0,678 | 0,083 | 0,222 | 0,941 | 0,532 | 0,232 | 0,425 | 0,816 | 0,875 | | 0,685 | 0,623 | 0,470 | 0,591 | 0,386 | 0,881 | 0,438 |
| APTT | 0,812 | 0,050 | 0,019 | 0,401 | 0,016 | 0,081 | 0,084 | 0,620 | 0,267 | 0,654 | 0,688 | 0,317 | 0,427 | 0,306 | 0,176 | 0,408 | 0,171 | 0,330 | 0,035 | 0,093 | 0,006 | 0,191 | 0,814 | 0,575 | 0,007 | 0,688 | 0,563 | 0,716 | 0,685 | | 0,760 | 0,258 | 0,033 | 0,545 | 0,198 | 0,284 |
| Fibrin | 0,949 | 0,412 | 0,255 | 0,870 | 0,324 | 0,047 | 0,565 | 0,183 | 0,915 | 0,740 | 0,838 | 0,013 | 0,138 | 0,461 | 0,013 | 0,230 | 0,201 | 0,325 | 0,344 | 0,182 | 0,227 | 0,218 | 0,023 | 0,006 | 0,447 | 0,257 | 0,026 | 0,552 | 0,623 | 0,760 | | 0,684 | 0,166 | 0,420 | 0,537 | 0,584 |
| DD | 1,000 | 0,181 | 0,001 | 0,874 | 0,237 | 0,733 | 0,043 | 0,027 | 0,094 | 0,019 | 0,010 | 0,169 | 0,701 | 0,480 | 0,723 | 0,211 | 0,063 | 0,914 | 0,604 | 0,202 | 0,094 | 0,785 | 0,949 | 0,793 | 0,029 | 0,956 | 0,034 | 0,904 | 0,470 | 0,258 | 0,684 | | 0,595 | 0,140 | 0,293 | 0,001 |
| PT | 0,835 | 0,416 | 0,608 | 0,788 | 0,248 | 0,595 | 0,261 | 0,100 | 0,505 | 0,634 | 0,765 | 0,726 | 0,131 | 0,396 | 0,993 | 0,634 | 0,953 | 0,234 | 0,169 | 0,579 | 0,084 | 0,873 | 0,006 | 0,554 | 0,406 | 0,485 | 0,548 | 0,094 | 0,591 | 0,033 | 0,166 | 0,595 | | 0,130 | 0,781 | 0,078 |
| Leukocytes | 0,205 | 0,381 | 0,150 | 0,403 | 0,604 | 0,405 | 0,157 | 0,717 | 0,266 | 0,127 | 0,936 | 0,147 | 0,821 | 0,596 | 0,725 | 0,184 | 0,351 | 0,642 | 0,954 | 0,342 | 0,248 | 0,149 | 0,297 | 0,384 | 0,177 | 0,388 | 0,792 | 0,625 | 0,386 | 0,545 | 0,420 | 0,140 | 0,130 | | 0,365 | 0,195 |
| PMNs | 0,756 | 0,124 | 0,029 | 0,559 | 0,526 | 0,624 | 0,055 | 0,622 | 0,570 | 0,597 | 0,116 | 0,521 | 0,398 | 0,451 | 0,124 | 0,364 | 0,755 | 0,436 | 0,131 | 0,070 | 0,820 | 0,873 | 0,426 | 0,360 | 0,911 | 0,918 | 0,331 | 0,300 | 0,881 | 0,198 | 0,537 | 0,293 | 0,781 | 0,365 | | 0,048 |
| Platelets | 0,907 | 0,139 | 0,002 | 0,258 | 0,200 | 0,855 | 0,015 | 0,419 | 0,384 | 0,364 | 0,304 | 0,550 | 0,993 | 0,277 | 0,920 | 0,242 | 0,357 | 0,744 | 0,990 | 0,535 | 0,388 | 0,887 | 0,821 | 0,784 | 0,523 | 0,437 | 0,816 | 0,406 | 0,438 | 0,284 | 0,584 | 0,001 | 0,078 | 0,195 | 0,048 | |

## Slide 3
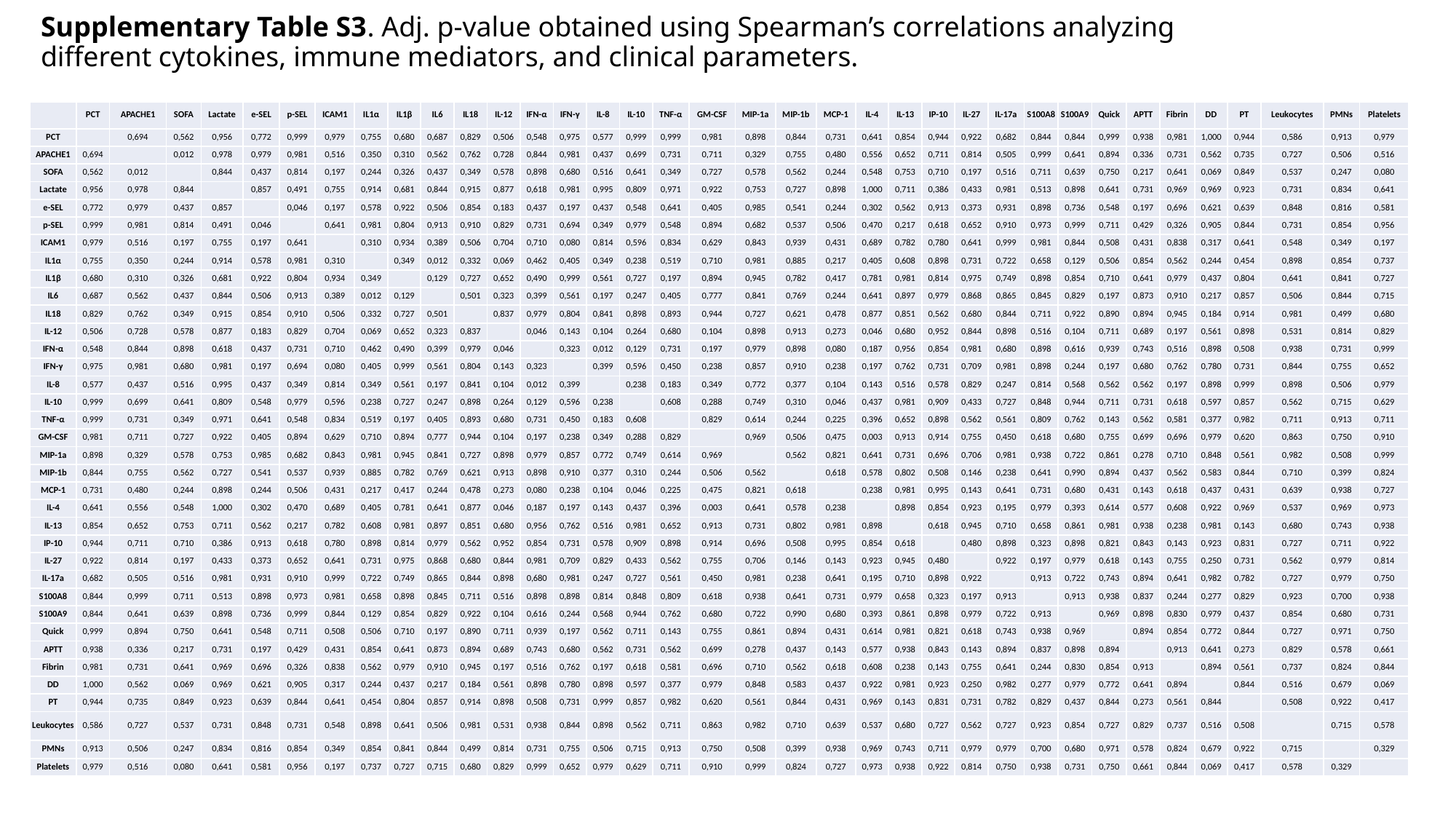

# Supplementary Table S3. Adj. p-value obtained using Spearman’s correlations analyzing different cytokines, immune mediators, and clinical parameters.
| | PCT | APACHE1 | SOFA | Lactate | e-SEL | p-SEL | ICAM1 | IL1α | IL1β | IL6 | IL18 | IL-12 | IFN-α | IFN-γ | IL-8 | IL-10 | TNF-α | GM-CSF | MIP-1a | MIP-1b | MCP-1 | IL-4 | IL-13 | IP-10 | IL-27 | IL-17a | S100A8 | S100A9 | Quick | APTT | Fibrin | DD | PT | Leukocytes | PMNs | Platelets |
| --- | --- | --- | --- | --- | --- | --- | --- | --- | --- | --- | --- | --- | --- | --- | --- | --- | --- | --- | --- | --- | --- | --- | --- | --- | --- | --- | --- | --- | --- | --- | --- | --- | --- | --- | --- | --- |
| PCT | | 0,694 | 0,562 | 0,956 | 0,772 | 0,999 | 0,979 | 0,755 | 0,680 | 0,687 | 0,829 | 0,506 | 0,548 | 0,975 | 0,577 | 0,999 | 0,999 | 0,981 | 0,898 | 0,844 | 0,731 | 0,641 | 0,854 | 0,944 | 0,922 | 0,682 | 0,844 | 0,844 | 0,999 | 0,938 | 0,981 | 1,000 | 0,944 | 0,586 | 0,913 | 0,979 |
| APACHE1 | 0,694 | | 0,012 | 0,978 | 0,979 | 0,981 | 0,516 | 0,350 | 0,310 | 0,562 | 0,762 | 0,728 | 0,844 | 0,981 | 0,437 | 0,699 | 0,731 | 0,711 | 0,329 | 0,755 | 0,480 | 0,556 | 0,652 | 0,711 | 0,814 | 0,505 | 0,999 | 0,641 | 0,894 | 0,336 | 0,731 | 0,562 | 0,735 | 0,727 | 0,506 | 0,516 |
| SOFA | 0,562 | 0,012 | | 0,844 | 0,437 | 0,814 | 0,197 | 0,244 | 0,326 | 0,437 | 0,349 | 0,578 | 0,898 | 0,680 | 0,516 | 0,641 | 0,349 | 0,727 | 0,578 | 0,562 | 0,244 | 0,548 | 0,753 | 0,710 | 0,197 | 0,516 | 0,711 | 0,639 | 0,750 | 0,217 | 0,641 | 0,069 | 0,849 | 0,537 | 0,247 | 0,080 |
| Lactate | 0,956 | 0,978 | 0,844 | | 0,857 | 0,491 | 0,755 | 0,914 | 0,681 | 0,844 | 0,915 | 0,877 | 0,618 | 0,981 | 0,995 | 0,809 | 0,971 | 0,922 | 0,753 | 0,727 | 0,898 | 1,000 | 0,711 | 0,386 | 0,433 | 0,981 | 0,513 | 0,898 | 0,641 | 0,731 | 0,969 | 0,969 | 0,923 | 0,731 | 0,834 | 0,641 |
| e-SEL | 0,772 | 0,979 | 0,437 | 0,857 | | 0,046 | 0,197 | 0,578 | 0,922 | 0,506 | 0,854 | 0,183 | 0,437 | 0,197 | 0,437 | 0,548 | 0,641 | 0,405 | 0,985 | 0,541 | 0,244 | 0,302 | 0,562 | 0,913 | 0,373 | 0,931 | 0,898 | 0,736 | 0,548 | 0,197 | 0,696 | 0,621 | 0,639 | 0,848 | 0,816 | 0,581 |
| p-SEL | 0,999 | 0,981 | 0,814 | 0,491 | 0,046 | | 0,641 | 0,981 | 0,804 | 0,913 | 0,910 | 0,829 | 0,731 | 0,694 | 0,349 | 0,979 | 0,548 | 0,894 | 0,682 | 0,537 | 0,506 | 0,470 | 0,217 | 0,618 | 0,652 | 0,910 | 0,973 | 0,999 | 0,711 | 0,429 | 0,326 | 0,905 | 0,844 | 0,731 | 0,854 | 0,956 |
| ICAM1 | 0,979 | 0,516 | 0,197 | 0,755 | 0,197 | 0,641 | | 0,310 | 0,934 | 0,389 | 0,506 | 0,704 | 0,710 | 0,080 | 0,814 | 0,596 | 0,834 | 0,629 | 0,843 | 0,939 | 0,431 | 0,689 | 0,782 | 0,780 | 0,641 | 0,999 | 0,981 | 0,844 | 0,508 | 0,431 | 0,838 | 0,317 | 0,641 | 0,548 | 0,349 | 0,197 |
| IL1α | 0,755 | 0,350 | 0,244 | 0,914 | 0,578 | 0,981 | 0,310 | | 0,349 | 0,012 | 0,332 | 0,069 | 0,462 | 0,405 | 0,349 | 0,238 | 0,519 | 0,710 | 0,981 | 0,885 | 0,217 | 0,405 | 0,608 | 0,898 | 0,731 | 0,722 | 0,658 | 0,129 | 0,506 | 0,854 | 0,562 | 0,244 | 0,454 | 0,898 | 0,854 | 0,737 |
| IL1β | 0,680 | 0,310 | 0,326 | 0,681 | 0,922 | 0,804 | 0,934 | 0,349 | | 0,129 | 0,727 | 0,652 | 0,490 | 0,999 | 0,561 | 0,727 | 0,197 | 0,894 | 0,945 | 0,782 | 0,417 | 0,781 | 0,981 | 0,814 | 0,975 | 0,749 | 0,898 | 0,854 | 0,710 | 0,641 | 0,979 | 0,437 | 0,804 | 0,641 | 0,841 | 0,727 |
| IL6 | 0,687 | 0,562 | 0,437 | 0,844 | 0,506 | 0,913 | 0,389 | 0,012 | 0,129 | | 0,501 | 0,323 | 0,399 | 0,561 | 0,197 | 0,247 | 0,405 | 0,777 | 0,841 | 0,769 | 0,244 | 0,641 | 0,897 | 0,979 | 0,868 | 0,865 | 0,845 | 0,829 | 0,197 | 0,873 | 0,910 | 0,217 | 0,857 | 0,506 | 0,844 | 0,715 |
| IL18 | 0,829 | 0,762 | 0,349 | 0,915 | 0,854 | 0,910 | 0,506 | 0,332 | 0,727 | 0,501 | | 0,837 | 0,979 | 0,804 | 0,841 | 0,898 | 0,893 | 0,944 | 0,727 | 0,621 | 0,478 | 0,877 | 0,851 | 0,562 | 0,680 | 0,844 | 0,711 | 0,922 | 0,890 | 0,894 | 0,945 | 0,184 | 0,914 | 0,981 | 0,499 | 0,680 |
| IL-12 | 0,506 | 0,728 | 0,578 | 0,877 | 0,183 | 0,829 | 0,704 | 0,069 | 0,652 | 0,323 | 0,837 | | 0,046 | 0,143 | 0,104 | 0,264 | 0,680 | 0,104 | 0,898 | 0,913 | 0,273 | 0,046 | 0,680 | 0,952 | 0,844 | 0,898 | 0,516 | 0,104 | 0,711 | 0,689 | 0,197 | 0,561 | 0,898 | 0,531 | 0,814 | 0,829 |
| IFN-α | 0,548 | 0,844 | 0,898 | 0,618 | 0,437 | 0,731 | 0,710 | 0,462 | 0,490 | 0,399 | 0,979 | 0,046 | | 0,323 | 0,012 | 0,129 | 0,731 | 0,197 | 0,979 | 0,898 | 0,080 | 0,187 | 0,956 | 0,854 | 0,981 | 0,680 | 0,898 | 0,616 | 0,939 | 0,743 | 0,516 | 0,898 | 0,508 | 0,938 | 0,731 | 0,999 |
| IFN-γ | 0,975 | 0,981 | 0,680 | 0,981 | 0,197 | 0,694 | 0,080 | 0,405 | 0,999 | 0,561 | 0,804 | 0,143 | 0,323 | | 0,399 | 0,596 | 0,450 | 0,238 | 0,857 | 0,910 | 0,238 | 0,197 | 0,762 | 0,731 | 0,709 | 0,981 | 0,898 | 0,244 | 0,197 | 0,680 | 0,762 | 0,780 | 0,731 | 0,844 | 0,755 | 0,652 |
| IL-8 | 0,577 | 0,437 | 0,516 | 0,995 | 0,437 | 0,349 | 0,814 | 0,349 | 0,561 | 0,197 | 0,841 | 0,104 | 0,012 | 0,399 | | 0,238 | 0,183 | 0,349 | 0,772 | 0,377 | 0,104 | 0,143 | 0,516 | 0,578 | 0,829 | 0,247 | 0,814 | 0,568 | 0,562 | 0,562 | 0,197 | 0,898 | 0,999 | 0,898 | 0,506 | 0,979 |
| IL-10 | 0,999 | 0,699 | 0,641 | 0,809 | 0,548 | 0,979 | 0,596 | 0,238 | 0,727 | 0,247 | 0,898 | 0,264 | 0,129 | 0,596 | 0,238 | | 0,608 | 0,288 | 0,749 | 0,310 | 0,046 | 0,437 | 0,981 | 0,909 | 0,433 | 0,727 | 0,848 | 0,944 | 0,711 | 0,731 | 0,618 | 0,597 | 0,857 | 0,562 | 0,715 | 0,629 |
| TNF-α | 0,999 | 0,731 | 0,349 | 0,971 | 0,641 | 0,548 | 0,834 | 0,519 | 0,197 | 0,405 | 0,893 | 0,680 | 0,731 | 0,450 | 0,183 | 0,608 | | 0,829 | 0,614 | 0,244 | 0,225 | 0,396 | 0,652 | 0,898 | 0,562 | 0,561 | 0,809 | 0,762 | 0,143 | 0,562 | 0,581 | 0,377 | 0,982 | 0,711 | 0,913 | 0,711 |
| GM-CSF | 0,981 | 0,711 | 0,727 | 0,922 | 0,405 | 0,894 | 0,629 | 0,710 | 0,894 | 0,777 | 0,944 | 0,104 | 0,197 | 0,238 | 0,349 | 0,288 | 0,829 | | 0,969 | 0,506 | 0,475 | 0,003 | 0,913 | 0,914 | 0,755 | 0,450 | 0,618 | 0,680 | 0,755 | 0,699 | 0,696 | 0,979 | 0,620 | 0,863 | 0,750 | 0,910 |
| MIP-1a | 0,898 | 0,329 | 0,578 | 0,753 | 0,985 | 0,682 | 0,843 | 0,981 | 0,945 | 0,841 | 0,727 | 0,898 | 0,979 | 0,857 | 0,772 | 0,749 | 0,614 | 0,969 | | 0,562 | 0,821 | 0,641 | 0,731 | 0,696 | 0,706 | 0,981 | 0,938 | 0,722 | 0,861 | 0,278 | 0,710 | 0,848 | 0,561 | 0,982 | 0,508 | 0,999 |
| MIP-1b | 0,844 | 0,755 | 0,562 | 0,727 | 0,541 | 0,537 | 0,939 | 0,885 | 0,782 | 0,769 | 0,621 | 0,913 | 0,898 | 0,910 | 0,377 | 0,310 | 0,244 | 0,506 | 0,562 | | 0,618 | 0,578 | 0,802 | 0,508 | 0,146 | 0,238 | 0,641 | 0,990 | 0,894 | 0,437 | 0,562 | 0,583 | 0,844 | 0,710 | 0,399 | 0,824 |
| MCP-1 | 0,731 | 0,480 | 0,244 | 0,898 | 0,244 | 0,506 | 0,431 | 0,217 | 0,417 | 0,244 | 0,478 | 0,273 | 0,080 | 0,238 | 0,104 | 0,046 | 0,225 | 0,475 | 0,821 | 0,618 | | 0,238 | 0,981 | 0,995 | 0,143 | 0,641 | 0,731 | 0,680 | 0,431 | 0,143 | 0,618 | 0,437 | 0,431 | 0,639 | 0,938 | 0,727 |
| IL-4 | 0,641 | 0,556 | 0,548 | 1,000 | 0,302 | 0,470 | 0,689 | 0,405 | 0,781 | 0,641 | 0,877 | 0,046 | 0,187 | 0,197 | 0,143 | 0,437 | 0,396 | 0,003 | 0,641 | 0,578 | 0,238 | | 0,898 | 0,854 | 0,923 | 0,195 | 0,979 | 0,393 | 0,614 | 0,577 | 0,608 | 0,922 | 0,969 | 0,537 | 0,969 | 0,973 |
| IL-13 | 0,854 | 0,652 | 0,753 | 0,711 | 0,562 | 0,217 | 0,782 | 0,608 | 0,981 | 0,897 | 0,851 | 0,680 | 0,956 | 0,762 | 0,516 | 0,981 | 0,652 | 0,913 | 0,731 | 0,802 | 0,981 | 0,898 | | 0,618 | 0,945 | 0,710 | 0,658 | 0,861 | 0,981 | 0,938 | 0,238 | 0,981 | 0,143 | 0,680 | 0,743 | 0,938 |
| IP-10 | 0,944 | 0,711 | 0,710 | 0,386 | 0,913 | 0,618 | 0,780 | 0,898 | 0,814 | 0,979 | 0,562 | 0,952 | 0,854 | 0,731 | 0,578 | 0,909 | 0,898 | 0,914 | 0,696 | 0,508 | 0,995 | 0,854 | 0,618 | | 0,480 | 0,898 | 0,323 | 0,898 | 0,821 | 0,843 | 0,143 | 0,923 | 0,831 | 0,727 | 0,711 | 0,922 |
| IL-27 | 0,922 | 0,814 | 0,197 | 0,433 | 0,373 | 0,652 | 0,641 | 0,731 | 0,975 | 0,868 | 0,680 | 0,844 | 0,981 | 0,709 | 0,829 | 0,433 | 0,562 | 0,755 | 0,706 | 0,146 | 0,143 | 0,923 | 0,945 | 0,480 | | 0,922 | 0,197 | 0,979 | 0,618 | 0,143 | 0,755 | 0,250 | 0,731 | 0,562 | 0,979 | 0,814 |
| IL-17a | 0,682 | 0,505 | 0,516 | 0,981 | 0,931 | 0,910 | 0,999 | 0,722 | 0,749 | 0,865 | 0,844 | 0,898 | 0,680 | 0,981 | 0,247 | 0,727 | 0,561 | 0,450 | 0,981 | 0,238 | 0,641 | 0,195 | 0,710 | 0,898 | 0,922 | | 0,913 | 0,722 | 0,743 | 0,894 | 0,641 | 0,982 | 0,782 | 0,727 | 0,979 | 0,750 |
| S100A8 | 0,844 | 0,999 | 0,711 | 0,513 | 0,898 | 0,973 | 0,981 | 0,658 | 0,898 | 0,845 | 0,711 | 0,516 | 0,898 | 0,898 | 0,814 | 0,848 | 0,809 | 0,618 | 0,938 | 0,641 | 0,731 | 0,979 | 0,658 | 0,323 | 0,197 | 0,913 | | 0,913 | 0,938 | 0,837 | 0,244 | 0,277 | 0,829 | 0,923 | 0,700 | 0,938 |
| S100A9 | 0,844 | 0,641 | 0,639 | 0,898 | 0,736 | 0,999 | 0,844 | 0,129 | 0,854 | 0,829 | 0,922 | 0,104 | 0,616 | 0,244 | 0,568 | 0,944 | 0,762 | 0,680 | 0,722 | 0,990 | 0,680 | 0,393 | 0,861 | 0,898 | 0,979 | 0,722 | 0,913 | | 0,969 | 0,898 | 0,830 | 0,979 | 0,437 | 0,854 | 0,680 | 0,731 |
| Quick | 0,999 | 0,894 | 0,750 | 0,641 | 0,548 | 0,711 | 0,508 | 0,506 | 0,710 | 0,197 | 0,890 | 0,711 | 0,939 | 0,197 | 0,562 | 0,711 | 0,143 | 0,755 | 0,861 | 0,894 | 0,431 | 0,614 | 0,981 | 0,821 | 0,618 | 0,743 | 0,938 | 0,969 | | 0,894 | 0,854 | 0,772 | 0,844 | 0,727 | 0,971 | 0,750 |
| APTT | 0,938 | 0,336 | 0,217 | 0,731 | 0,197 | 0,429 | 0,431 | 0,854 | 0,641 | 0,873 | 0,894 | 0,689 | 0,743 | 0,680 | 0,562 | 0,731 | 0,562 | 0,699 | 0,278 | 0,437 | 0,143 | 0,577 | 0,938 | 0,843 | 0,143 | 0,894 | 0,837 | 0,898 | 0,894 | | 0,913 | 0,641 | 0,273 | 0,829 | 0,578 | 0,661 |
| Fibrin | 0,981 | 0,731 | 0,641 | 0,969 | 0,696 | 0,326 | 0,838 | 0,562 | 0,979 | 0,910 | 0,945 | 0,197 | 0,516 | 0,762 | 0,197 | 0,618 | 0,581 | 0,696 | 0,710 | 0,562 | 0,618 | 0,608 | 0,238 | 0,143 | 0,755 | 0,641 | 0,244 | 0,830 | 0,854 | 0,913 | | 0,894 | 0,561 | 0,737 | 0,824 | 0,844 |
| DD | 1,000 | 0,562 | 0,069 | 0,969 | 0,621 | 0,905 | 0,317 | 0,244 | 0,437 | 0,217 | 0,184 | 0,561 | 0,898 | 0,780 | 0,898 | 0,597 | 0,377 | 0,979 | 0,848 | 0,583 | 0,437 | 0,922 | 0,981 | 0,923 | 0,250 | 0,982 | 0,277 | 0,979 | 0,772 | 0,641 | 0,894 | | 0,844 | 0,516 | 0,679 | 0,069 |
| PT | 0,944 | 0,735 | 0,849 | 0,923 | 0,639 | 0,844 | 0,641 | 0,454 | 0,804 | 0,857 | 0,914 | 0,898 | 0,508 | 0,731 | 0,999 | 0,857 | 0,982 | 0,620 | 0,561 | 0,844 | 0,431 | 0,969 | 0,143 | 0,831 | 0,731 | 0,782 | 0,829 | 0,437 | 0,844 | 0,273 | 0,561 | 0,844 | | 0,508 | 0,922 | 0,417 |
| Leukocytes | 0,586 | 0,727 | 0,537 | 0,731 | 0,848 | 0,731 | 0,548 | 0,898 | 0,641 | 0,506 | 0,981 | 0,531 | 0,938 | 0,844 | 0,898 | 0,562 | 0,711 | 0,863 | 0,982 | 0,710 | 0,639 | 0,537 | 0,680 | 0,727 | 0,562 | 0,727 | 0,923 | 0,854 | 0,727 | 0,829 | 0,737 | 0,516 | 0,508 | | 0,715 | 0,578 |
| PMNs | 0,913 | 0,506 | 0,247 | 0,834 | 0,816 | 0,854 | 0,349 | 0,854 | 0,841 | 0,844 | 0,499 | 0,814 | 0,731 | 0,755 | 0,506 | 0,715 | 0,913 | 0,750 | 0,508 | 0,399 | 0,938 | 0,969 | 0,743 | 0,711 | 0,979 | 0,979 | 0,700 | 0,680 | 0,971 | 0,578 | 0,824 | 0,679 | 0,922 | 0,715 | | 0,329 |
| Platelets | 0,979 | 0,516 | 0,080 | 0,641 | 0,581 | 0,956 | 0,197 | 0,737 | 0,727 | 0,715 | 0,680 | 0,829 | 0,999 | 0,652 | 0,979 | 0,629 | 0,711 | 0,910 | 0,999 | 0,824 | 0,727 | 0,973 | 0,938 | 0,922 | 0,814 | 0,750 | 0,938 | 0,731 | 0,750 | 0,661 | 0,844 | 0,069 | 0,417 | 0,578 | 0,329 | |
